# Genomic Insights into the Ancestry and Demographic History of South America

**DOI:** 10.1371/journal.pgen.1005602

**Published:** 2015-12-04

**Authors:** Julian R. Homburger, Andrés Moreno-Estrada, Christopher R. Gignoux, Dominic Nelson, Elena Sanchez, Patricia Ortiz-Tello, Bernardo A. Pons-Estel, Eduardo Acevedo-Vasquez, Pedro Miranda, Carl D. Langefeld, Simon Gravel, Marta E. Alarcón-Riquelme, Carlos D. Bustamante

**Affiliations:** 1 Department of Genetics, Stanford University, Stanford, California, United States of America; 2 Laboratorio Nacional de Genómica para la Biodiversidad (LANGEBIO), CINVESTAV, Irapuato, Guanajuato, Mexico; 3 McGill University and Genome Quebec Innovation Centre, Montreal, Quebec, Canada; 4 Department of Human Genetics, McGill University, Montreal, Quebec, Canada; 5 Arthritis and Clinical Immunology, Oklahoma Medical Research Foundation, Oklahoma City, Oklahoma, United States of America; 6 Sanatorio Parque, Rosario, Argentina; 7 Facultad de Medicina, Universidad Nacional Mayor de San Marcos, Hospital Nacional Guillermo Almenara Irigoyen, Lima, Peru; 8 Centro de Estudios Reumatologicos, Santiago, Chile; 9 Center for Public Health Genomics, Wake Forest School of Medicine, Winston-Salem, North Carolina, United States of America; 10 GENYO, Centre for Genomics and Oncological Research: Pfizer/ University of Granada/ Andalusian Regional Government, Granada, Spain; Universidade Federal de Minas Gerais, BRAZIL

## Abstract

South America has a complex demographic history shaped by multiple migration and admixture events in pre- and post-colonial times. Settled over 14,000 years ago by Native Americans, South America has experienced migrations of European and African individuals, similar to other regions in the Americas. However, the timing and magnitude of these events resulted in markedly different patterns of admixture throughout Latin America. We use genome-wide SNP data for 437 admixed individuals from 5 countries (Colombia, Ecuador, Peru, Chile, and Argentina) to explore the population structure and demographic history of South American Latinos. We combined these data with population reference panels from Africa, Asia, Europe and the Americas to perform global ancestry analysis and infer the subcontinental origin of the European and Native American ancestry components of the admixed individuals. By applying ancestry-specific PCA analyses we find that most of the European ancestry in South American Latinos is from the Iberian Peninsula; however, many individuals trace their ancestry back to Italy, especially within Argentina. We find a strong gradient in the Native American ancestry component of South American Latinos associated with country of origin and the geography of local indigenous populations. For example, Native American genomic segments in Peruvians show greater affinities with Andean indigenous peoples like Quechua and Aymara, whereas Native American haplotypes from Colombians tend to cluster with Amazonian and coastal tribes from northern South America. Using ancestry tract length analysis we modeled post-colonial South American migration history as the youngest in Latin America during European colonization (9–14 generations ago), with an additional strong pulse of European migration occurring between 3 and 9 generations ago. These genetic footprints can impact our understanding of population-level differences in biomedical traits and, thus, inform future medical genetic studies in the region.

## Author Summary

South America is home to over 400 million people who share a rich demographic history, including settlement by Native Americans, European colonization, and the African slave trade. We use genomic data to infer which populations from Europe and the Americas contributed to these admixture events. We provide evidence for multiple origins of the Native American ancestry of admixed South American Latinos. The Native American ancestral component correlates strongly with geography, indicating that admixture occurred between European colonists and local Native American populations throughout South America. We also show that the European ancestry of South American Latinos comes mainly from the Iberian peninsula, however, a significant number of Argentinians have European ancestry from other Southern European regions. The genetic signal of European admixture in South American populations is younger than the signal observed in Mexico and the Caribbean. We find evidence for a second pulse of European migration to many regions of South America subsequent to the original colonization. These results demonstrate the heterogeneous nature of the Latino population in South America and help elucidate the complex genetic and admixture events that shaped the population structure of the region.

## Introduction

Our understanding of fine-scale patterns of population structure in humans has dramatically increased with the advent and deployment of fast, inexpensive, and accurate genome-wide technologies for assaying variation [1–3]. However, our understanding of regional patterns of genomic variation is quite poor in many parts of the world particularly in populations that are currently underrepresented in GWAS studies, including those in Latin America [4]. Understanding patterns of genomic variation is especially important for populations throughout the Americas, which have undergone multiple recent admixture events, making the reconstruction of their evolutionary past and the design of multi-ethnic medical genetic studies challenging. Recently, studies in Mexico, the Caribbean, and throughout the Americas have shed light on the complex demographic processes that occurred in those regions and have illuminated how differences in the pre- and post-colonial history have shaped differences in genomic variation that ultimately impact variation in complex biomedical traits [5–7]. The South American landmass features unique geographic, archaeological, and historical records that are distinct from other regions of the Americas [8]. The contributions of these events to patterns of genomic variation remains to be laid out to a greater extent. For example, in contrast to North America, South America’s indigenous population history derives from a single migration wave that rapidly expanded southwards throughout the Andean highlands and eastwards into the Amazon basin [9]. Previous analyses of native South American variation based on microsatellites have reported a west-to-east difference in genetic diversity between Andean and eastern Brazilian tribes as one of the strongest signals of sub-continental genetic differentiation [10–12]. The largest human settlements in South America, however, occurred throughout the Andean region and likely represent a major source of Native American variation in present day South American Latinos. Characterizing the extent of substructure and differential contribution of these ancestral components is therefore crucial to understanding the genetic heterogeneity of the South American population.

Previous studies on South American Latino populations have either used a limited number of genetic markers to evaluate continental-level patterns of population structure or focused on particular geographic regions [13–18]. Many of these studies and others have demonstrated a large amount of genetic diversity in Native American and mestizo populations, especially between different geographic regions [11–13,19]. Wang et al. analyzed multiple mestizo populations throughout South America using 678 microsatellite markers [13] and found evidence of correlations between ancestry components and geography. The Galanter et. al. and Ruiz-Linares et al. studies [15,16] used a limited set of ancestry informative markers to analyze the global ancestry proportions throughout Latin America. However, due to the smaller numbers of markers, these studies were unable to perform analyses that rely upon dense genetic information such as local ancestry inference, ancestry specific principal components analysis, and demographic modeling based upon ancestry tract length. Recent work in Brazil using dense genomic information has demonstrated that individuals differ markedly in ancestry proportions both within and between populations in metropolitan regions of South America [20]. They also demonstrate significant variation within the European and African ancestry components.

Here, we expand upon previous work by focusing on admixed populations from five countries in Spanish speaking South America (Argentina, Chile, Colombia, Ecuador, and Peru), spanning much of the Andean region of the continent. Similar to other areas in Latin America, South America has experienced multiple migration and admixture events, including Native American settlement, European colonization, and the African slave trade. However, the timing and magnitude of migration waves from a myriad of continental and subcontinental ancestral groups varies dramatically throughout the continent and affects the population genetic profile of the region at a local scale.

The earliest settlements in South America date back over 14,000 years ago [8]. Native Americans developed multiple civilizations throughout the continent, including settlements in the Andes, the Amazon, and along the coasts. In the 16^th^ century, European colonization and conquest led to a dramatic population bottleneck in the Native American population as well as an increasing influx of European migrants, quickly followed by admixture with West Africans brought to the Americas through the slave trade. During the following centuries, there was continuous admixture between European, Native Americans, and African individuals. Early European migration into the Spanish South American colonies came mainly from the Iberian Peninsula. Spanish conquistadors in the early 16^th^ century conquered many of the indigenous populations in the Andean region of South America, establishing South American colonies throughout the continent [21]. All five of the countries studied here were originally part of the Spanish viceroyalty of Peru. These Spanish colonies followed separate but related developmental paths, eventually splitting into the viceroyalties of Peru, New Granada, and Rio de la Plata. The Peruvian colony was a major source of silver for the Spanish Empire, while the colonies in Rio de la Plata (including present day Argentina) and New Granada (including Colombia and Ecuador) became important commercial centers [21,22]. The Spanish colonies in South America continued to receive immigration from Europe concurrent with admixture with Native American populations. In the 19^th^ and 20^th^ centuries, there is evidence of increased migration from many regions of Southern Europe, especially in Argentina [23,24].

To explore the impact of this complex demographic history upon the current genetic structure of South American Latino populations, we analyze single nucleotide polymorphism (SNP) genotyping data from 436 unrelated admixed samples, including 175 Argentinians, 119 Peruvians, 27 Chileans, 19 Ecuadorians, and 96 Colombians. We combined these data with reference panels of European and Native American populations, and applied admixture deconvolution methods to trace back the origin of each ancestry component within Europe and the Americas. We also analyzed the length distribution of ancestral segments in admixed individuals to test hypotheses about past migration patterns and examine whether different countries have experienced different genetic histories.

## Results and Discussion

### Global ancestry composition

**Fig 1:**
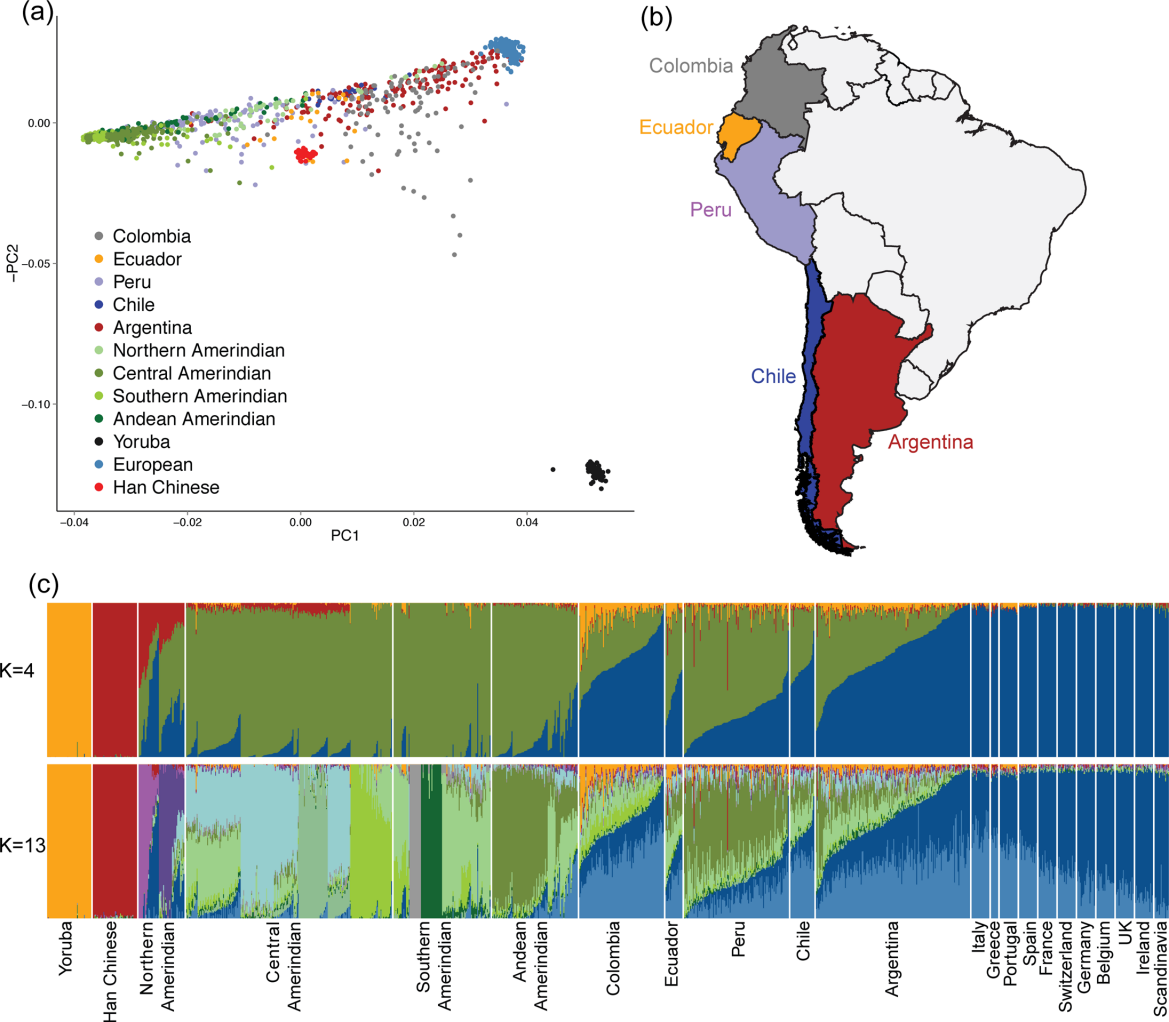
Global ancestry analysis of South American populations. (a) Principal Components Analysis of admixed individuals and continental reference panels. Each individual is represented as a point colored by country, region, or continent of origin. (b) Map of sampled populations. Countries of origin for admixed South Americans are highlighted and colored as in (a). (c) ADMIXTURE plot of admixed individuals and continental reference panels. Each individual is represented as a thin vertical bar. The colors represent the proportion of ancestry assigned to each cluster for each individual. *K* = 4 and *K* = 13 models are shown above, *K* = 2 through *K* = 15 models are available in S4 and S5 Figs.

To characterize the ancestral components of South American Latino individuals from Colombia, Ecuador, Peru, Chile, and Argentina, we applied unsupervised clustering models and principal components analysis to genotype data from ancestral and admixed populations (Fig 1) (see Methods). This data set contains 436 admixed South American individuals together with 204 European individuals from the POPRES study [1], 50 Yoruban and 50 Han Chinese from the 1000 Genomes Project [3], and 493 unmasked Native American individuals from Reich *et al*. 2012 [9]. The South American individuals showed varying proportions of European, Native American and, to a lesser extent, West African ancestry in PCA space, supporting the notion of a broad range of global ancestry patterns throughout South America. We observed some dispersion of Native American individuals away from the main ancestral cluster due to the presence of European admixture.

We then ran clustering models for *K* = 2 through *K* = 15 ancestral populations with ADMIXTURE [25] on a total of 1,233 individuals. Cross validation errors for the ADMIXTURE analysis are shown in S2 Fig. The minimum CV error was observed at K = 13. When clustering is performed assuming *K* = 4 ancestral populations (Fig 1C), the algorithm separates the individuals into four major continental clusters. Average continental ancestry proportions for each of the admixed populations are shown in Table 1. As expected from historical records [21,22] and previous results from other Latino populations in the Caribbean [6] and Mexico [5], South American Latino individuals show a mixture of European, Native American, and African ancestry. However, some populations, especially those in Peru, Chile, and Argentina, tend to have a smaller proportion of African ancestry than seen in Latino populations in the Caribbean (p < 2.2 x 10^−16^, Wilcoxon test, S6 Fig), also observed in previous analyses [13,16–18]. We find significant differences in global ancestry proportions between countries within South America. The Peruvian individuals tend to have a higher proportion of Native American ancestry than individuals from any of the other South American populations (Tukey HSD Test, p < 0.001 vs. Argentina, Chile, Colombia, Ecuador; S3 Fig). We observed multiple Peruvian individuals with a >25% proportion of East Asian ancestry, which is not surprising given that there were large Asian migrations to Peru especially during the 19^th^ and early 20^th^ century where laborers from Guandong (formerly Canton) province in China were brought to the country [26]. Peru opened its borders to Asian immigration in 1849, and it is estimated that over 87,000 Chinese individuals entered Peru between 1859 and 1874 [22]. This East Asian ancestry component is also seen in the Northern Amerindian individuals. These individuals are from Eskimo, Aleut, and Na-Dene populations and the observed clustering is consistent with the hypothesis of multiple waves of gene flow from Asia to America suggested by a previous study [9]. At higher values of K in ADMIXTURE, these individuals are assigned to their own ADMIXTURE component, indicating a unique ancestry component that is separate from the East Asian cluster (Fig 1 and S5 Fig).

**Table 1:** Global ancestry proportions estimated through ADMIXTURE K = 4.

Population	European	Native American	West African	East Asian
Argentina	0.673	0.277	0.036	0.014
Chile	0.572	0.387	0.025	0.017
Colombia	0.625	0.274	0.092	0.009
Ecuador	0.408	0.501	0.068	0.023
Peru	0.260	0.683	0.032	0.025

The Argentinian population has a significantly higher proportion of European ancestry than the Peruvian, Chilean, and Ecuadorian populations (Tukey HSD Test, p = 0.018 vs. Chile, p = 0.129 vs. Colombia, p<0.001 vs. Peru and Ecuador) with some individuals having close to 100% European ancestry (S3 Fig). Even so, there is a large range of ancestry proportions within individuals from Argentina, consistent with previous results based on a small number of ancestry informative markers and blood group antigens [17,27,28]. This variance is most likely a result of the contrasting histories of different Argentinean regions. For example, the original Spanish settlers of Argentina came through the Pacific/Andean region [21]. However, as Argentina developed, individuals from Spain and Southern Europe settled throughout the coastal regions on the Atlantic [23]. We also observed a small number of Argentinian individuals with relatively high amounts of African ancestry, whereas the rest of the individuals have a very low African ancestry component. This diversity is reflected in the large range in ancestry proportions seen within Argentina and is consistent with previous studies [17,28,29].

At higher order *K*s (*K* = 13 in Fig 1), we observed significant substructure in both the Native American and European populations. The North-South gradient among European populations is strongly correlated with the latitude of each country’s capital (p < 2.2 X 10^−16^, linear regression), with a southern European component (light blue) most prominent in Spain, Portugal, Italy, and Greece. Most of the admixed Latino individuals in the sample have a high proportion of this southern European component, suggesting that the Europeans involved in admixture events in South America are from the Iberian Peninsula and Mediterranean Europe. This observation is consistent with historical migration patterns and maintained cultural influence [19]. On the other hand, the primary cluster of Native ancestry is reflective of the local indigenous diversity. We find that a component of the Native American ancestry in the Peruvian samples is shared with local Andean native groups, such as Quechua and Aymara, and that of Colombians is more closely shared with the Southern and Central Amerindian groups (Fig 1, K = 13). In contrast, we see that the Native American component in Argentina and Chile is shared between components from Central/Southern Native American and Andean Native American groups, showing a wider range of ancestral origins that we explore below in further analyses (Fig 1C, K = 13).

Sex biased ancestry is an important feature of many Latin American populations, and has been observed and described thoroughly in many previous research articles [6,18]. European migrants to the Americas were mainly male, especially during the earlier years of colonization. This has resulted in increased Amerindian ancestry on the X-chromosome when compared to the autosomes. After excluding admixed males from the analysis, we had admixed individuals from only four populations: Argentina, Chile, Colombia, and Peru. We compared ADMIXTURE estimates at K = 3 of autosomal and X-chromosomal ancestry (S7 Fig). We find an increase in Native American ancestry on the X-chromosome compared to the autosomes (S8 Fig, Wilcoxon p < 0.001). This is suggestive of the fact that there was an overabundance of European males and Amerindian females that participated in the admixture process.

### Subcontinental ancestry components in South America

To identify the admixed individuals’ subcontinental lineages rooted within Europe and South America, we performed ancestry-specific PCA analysis. ASPCA is a technique developed to perform principal components analysis on the fraction of an individual’s ancestry from a specific continental origin. In contrast to PCA, which is performed on individual (unphased) diploid genotype calls, ASPCA is performed on phased haploid genomes conditional on ancestry calls (see Methods for details).

**Fig 2:**
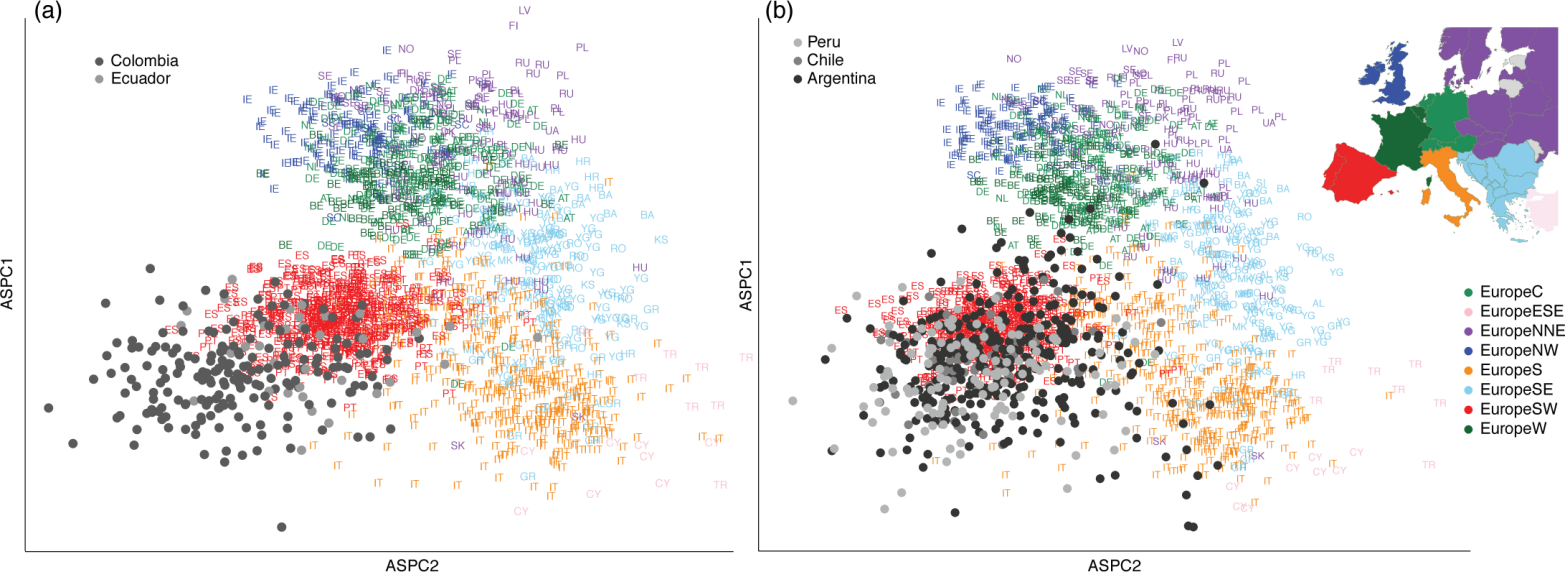
European ancestry specific analysis. (a) European Ancestry Specific PCA of haploid genomes from Colombia and Ecuador with greater than 25% estimated European ancestry combined with 2,882 haploid genomes from the POPRES data set. Admixed Latino individuals are shown in shades of grey, while European individuals are colored according to region and represented as a two-character country code. (b) European Ancestry Specific PCA of haploid genomes from Peru, Chile, and Argentina with greater than 25% estimated European ancestry combined with the same reference European data set as in (a). The inset map shows the color-coded regions within Europe of the POPRES reference panel. To maximize SNP overlap between data sets, ASPCA analyses were performed separately for each subset of South American Latino populations (see Methods).

To explore their European origins, we combined our admixed individuals with the POPRES European data set [1] and performed ASPCA on the merged data set (Fig 2). Due to the limited overlap between the POPRES data set and our samples, we performed ASPCA on the Argentinian, Chilean, and Peruvian haplotypes separately from the Colombian and Ecuadorian haplotypes. The European reference samples cluster according to geography [30]. We find that the majority of the European haplotypes of the admixed samples cluster with Iberian and Southern Europeans, consistent with historical records and previous reports [6,31]. However, we observed interesting differences between countries in South America. For example, Argentina showed the highest number of European haplotypes that cluster in the Italian peninsula. This is consistent with recent migration events from Italy to Argentina in the late 19^th^ and early 20^th^ centuries [22]. Between 1880 and 1930, 2.3 million of the 4.7 million migrants to Argentina had Italian nationality [24]. We also find that Argentina has the largest range in the European ancestry components and even includes two haploid genomes that cluster near individuals from Germany, Poland, and Hungary in the top right of the ASPCA plot (Fig 2). No other South American population showed samples with such distant ancestry from the Iberian cluster, nor other Latino samples from previous studies in the Caribbean and Mexico [5,6].

To further our investigation of the European component beyond the Spanish ancestry found in the Iberian Peninsula, we combined masked samples from the Canary Islands with South American individuals from Colombia and Ecuador. The Canary Islands were colonized by the Spanish in the early 15^th^ century and became a stopping point for Spanish on their way to the Americas. Here, we find undifferentiated patterns of ancestry between the European component of these three populations (S9 Fig), suggesting that the European ancestry of these groups either originated from a similar source on the Iberian peninsula or that methods of increased resolution are needed to untangle more subtle differences.

To investigate the Native American component of the South American individuals’ ancestry, we combined our samples with those from 49 Native American populations previously genotyped [9]. We removed Native American samples that appeared as outliers in ASPCA space and that were geographically distant from South America (see Methods and S10 Fig). We also excluded Native American individuals with greater than 10% estimated European ancestry, as we found these individuals were biasing the principal components analysis towards a European/Native American axis (S11, S12 and S13 Figs). For visualization purposes, Native American populations were grouped corresponding to the labels used in Reich *et al*. [9] and are referenced geographically (see S1 Table for mapping).

**Fig 3:**
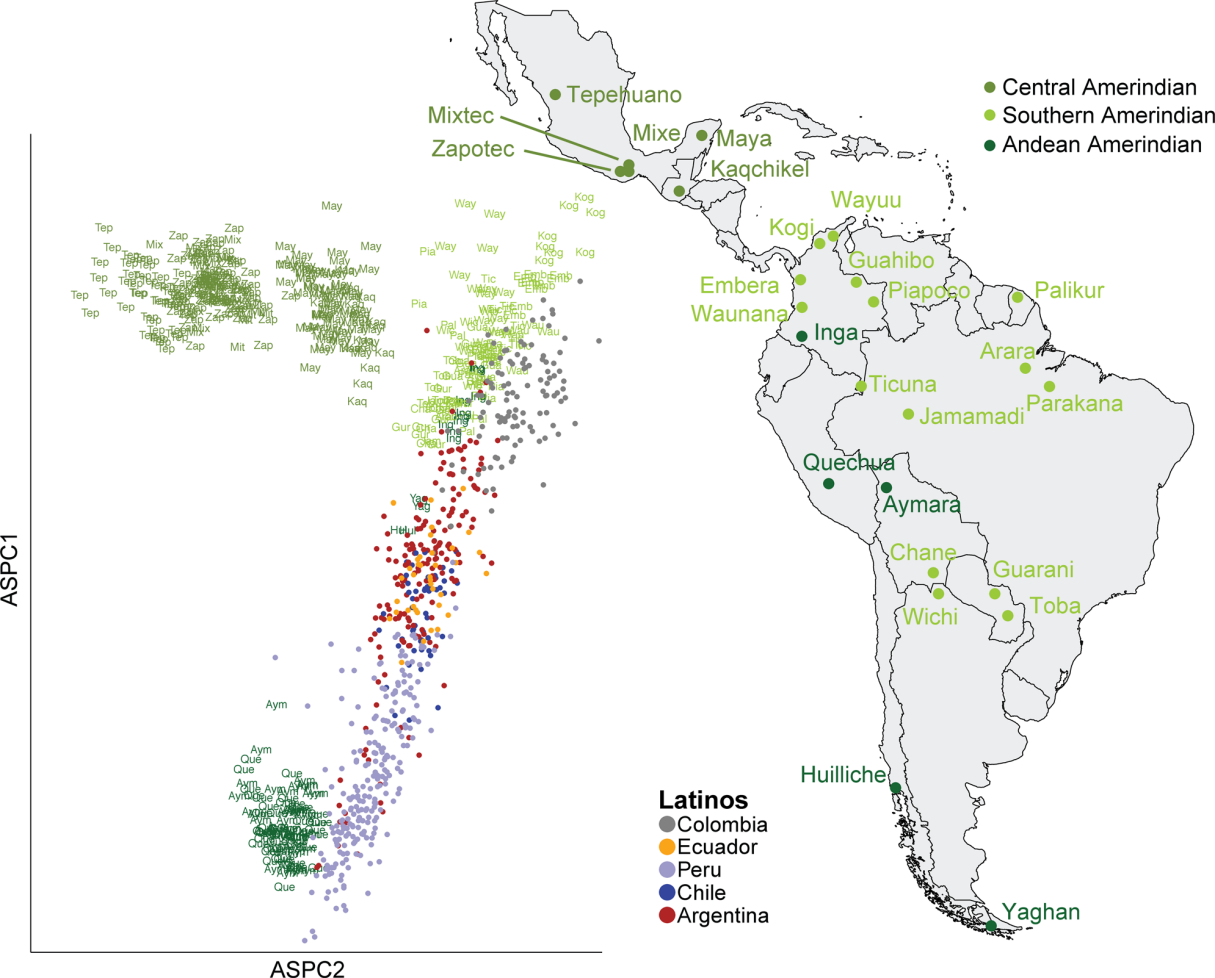
Native American ancestry specific analysis. Native American Ancestry Specific PCA of all Latino haploid genomes with greater than 25% estimated Native American ancestry. Each masked haploid genome from admixed individuals is represented by a single point colored by population of origin. Native American haploid genomes are plotted as the first three letters of the population name and colored according to the regional groupings. The approximate sampling location for each of the Native American parental populations is shown on the map of Latin America.

We find that the Native American component of the South American haplotypes clusters along a gradient between the Andean Amerindian populations and the Southern Amerindian populations along ASPC1 and ASPC2 (Fig 3). Notably, the Native American ancestry in the admixed South American individuals is drastically different from the genetic components observed among Central and Northern Native American groups, such as Kaqchikel in Guatemala and Zapotec or Tepehuano in Mexico. None of these groups showed close affinities with Latino-derived South American haplotypes, supporting the notion of a highly substructured architecture of the Native American component among Latinos from different regions across Latin America.

Our ASPCA analysis revealed that South American native haplotypes cluster primarily into two groups: one represented by central Andean individuals, such as Quechua and Aymara, and another group that includes most of the remaining native populations from South America. The differentiation between the Andean Amerindians and other South Amerindians is consistent with previous results using Y chromosome and mtDNA analyses [11,12], and suggests that the mountain range of the Andes acted as a major geographic barrier to gene flow during Native American evolution. This created further population structure among South Native American groups separating populations in the Amazon and east coastal regions from highland populations in the Andes. Interestingly, a number of the populations classified as Andean such as the Hulliche, Inga, and Yahgan clustered close to the Southern/Amazonian Native Americans and far from the other Andean Native Americans such as the Quechua and Aymara. Reich *et al*. in 2012 [9] suggested that, based on linguistic affinities, these populations would be expected to cluster with the Aymara and Quechua populations. Indeed, among the samples from the main Amazonian cluster in Fig 3, these are the only ones spreading towards the Quechua/Aymara cluster, supporting the idea of pre-Columbian admixture events giving rise to populations like the Inga, Huilliche, and Yahgan along the Andes. The separation between the Andean and other South American populations is consistent with the hypotheses of either multiple migration routes into South America, with an early split soon after crossing the Isthmus of Panama, or restricted levels of gene flow shortly after establishment of Native American settlements in the continent [8,11,12,32]. Likewise, the clustering of northern Argentinian Wichi and Paraguayan Guarani and Toba with lowland groups from Brazil and Colombia, suggests an Amazonian origin of Native American migration into the Gran Chaco and Pampas areas rather than strong evidence of a trans-Andean route. The branching pattern of these ancestral migrations have directly impacted the genetic profile of present day South American Latino populations, even between neighboring countries such as Argentina and Chile. We detail these patterns in what follows.

The clustering of the masked haploid genomes from the admixed individuals tended to be population specific (Fig 3). We find that the Peruvian individuals cluster more closely with the Andean Native American individuals than with any other Native American group, suggesting that the Native American component of the Peruvian population is mainly from the Andean region. While the Andean Native Americans and Peruvian individuals cluster closely, many of them do not overlap. Both the Quechua and Aymara individuals are from the Central Andes, while the admixed individuals are from Lima. Mitochondrial and Y-chromosomal studies of Andean ancestry have indicated that there is relatively low geographically-correlated genetic diversity in the Andean region, likely due to the historically higher gene flow and population size in the Andean region [11,12]. While there seems to be less geographic correlation in ancestry in the Andean Native Americans than in other Native American populations, some geographic stratification may be detected through high-density genotyping that was not detected using mitochondrial or Y-chromosomal analysis. In other populations with lower levels of genetic differentiation, such as Europeans, high density genotyping data revealed correlations between geography and ancestry [30]. Also, our reference panel has little representation from coastal Peruvian Native Americans, and these groups may also have contributed to the admixture process in cosmopolitan areas.

Argentinian individuals show a broader range of indigenous ancestry: some cluster closer to the Southern/Amazonian Native Americans, while others cluster with the Latino Peruvians and the Andean group, reflecting a rich diversity of pre-Columbian roots in Argentina, whose geography spans the breadth of the continent from the Andes to the Atlantic, thus absorbing haplotypes from both major streams of Native American migration. We find only a marginal relationship between clustering and sampling location within Argentina. We find that sampling latitude is marginally associated with ASPC1 in a linear regression (p = 0.025, S14 Fig), and not significantly associated with ASPC2 (p = 0.3387, S15 Fig). We find no significant linear relationship between longitude and ASPCs (p = 0.322 vs. ASPC1 and p = 0.844 vs. ASPC2; S16 and S17 Figs). However, we do not expect the sampling locations in our current sample to be indicative of an individual’s history to this degree of resolution. Most individuals were sampled at hospitals in major cities throughout Argentina, with the largest number of individuals sampled in Buenos Aires. Because of recent major migrations of individuals, especially from rural to urban areas, current location may not be indicative of the location of an individual’s ancestors. There has also been recent intraregional migration throughout South America, especially in urban regions such as Buenos Aires [33,34]. This could be contributing to the genetic diversity we observe within the Argentinian individuals’ Native American ancestry. A sampling scheme based upon the “four grandparent” ancestry principle, such as the one used in the European POPRES [1] study, along with more representation for different regions throughout Argentina may better elucidate finer scale structure in the country, although this is also known to be imperfect [17].

In contrast, the Colombian and Ecuadorian Latino haplotypes tend to cluster with geographically nearby Southern Native Americans, such as the Wayuu, Piapoco, and Ticuna from Colombia. The Ecuadorian individuals cluster farther away from this ancestral group than the Colombians, which could be due to the lack of Ecuadorian Native American groups in the reference panel or be the result of admixture with Andean Native American lineages.

The Chilean individuals cluster towards the middle of the admixed group, between the Andean cluster and the Chilean Huilliche and Yaghan samples. The Native American reference panel used here does not include many Native Americans from Southern Chile. Only two haploid genomes from one Hulliche individual are in the subset of the reference panel used for analysis due to the high proportion of European ancestry in the remaining Hulliche individuals. The lack of representation of Hulliche and other Chilean Native Americans could explain why we do not see a strong differentiation of the admixed Chilean haploid genomes. A deeper sampling effort is needed to assess fine-scale genetic patterns within Chile. We find that the Native American ancestry of admixed Latinos is associated with population of origin (ANOVA p < 2 x 10^−16^ for both ASPC1 and ASPC2). This is consistent with many previous results in population history analysis, which have also shown strong correlation between geographic features and ancestry.

To further investigate the differences between the European and Native American ancestry components of South American individuals, we used GERMLINE [35] to identify genomic regions of identity by descent (IBD) in the admixed individuals and compared the patterns of IBD matching within and among populations to the local ancestry calls inferred throughout each IBD match. We find 12,348 segments of IBD shared within populations compared with only 4,941 segments of IBD shared between populations. On average, the individuals from Colombia share the most IBD within the population (15.2 cM), followed by Chile (3.42 cM), Ecuador (2.58 cM), Peru (2.06 cM), and Argentina (0.84 cM). We find that segments shared between populations are shorter than those shared within populations (Wilcox p < 2.2e-16). For IBD segments that could be identified using a haploid comparison, we calculated for each segment the proportion of European, Native American, and African local ancestry. We find that in both within and between populations, longer IBD segments have a higher proportion of European ancestry (linear regression, p = 4.04 x 10^−16^). The effect size based upon linear regression is greater in IBD segments shared between populations (β = 0.050 ± 0.0084 s.e., p = 3.7 x 10^−9^) than in IBD segments shared within populations (β = 0.0076 ± 0.0011 s.e., p = 4.4 x 10^−11^) (S18, S19 and S20 Figs). To test if a single admixed population primarily drove the observed effect, we also analyzed the shared IBD segments within each admixed South American population on its own. We observed a significant association in a linear regression model between IBD segment length and ancestry in the Peruvian and Argentinian populations (Peru p = 6.01 x 10^−8^, Argentina p = 0.0088, S21 Fig), a suggestive association in the Colombian populations (p = 0.0955, S21 Fig), and found no evidence for the association in Chilean or Ecuadorian populations (Chile p = 0.132, Ecuador p = 0.903, S21 Fig). However, the difference in significance may be a function of the differential power to detect this effect within populations due to sample sizes differences, since we have the lowest number of admixed individuals from Chile and Ecuador.

We also find that the majority of the long IBD segments (greater than 10 cM) shared between populations are of European ancestry (20 European vs. 2 Native American). IBD tracts shared between populations tend to consist of only a single continental ancestry, while those shared within populations often contain multiple ancestry switch points. These patterns are consistent with previous studies of IBD sharing in Latino populations [36] and suggest that the most recent common ancestors shared between Latino populations in South America are much more likely to be European than Native American, and that these trace back to a reduced source of founders. These results are also supported by the ASPCA analyses, where we find remarkable substructure among the different Native American components of each Latino population, but similar patterns of Southern European ancestry across Latino populations in ASPCA space.

To further confirm the results of the ASPCA analysis, we looked for diploid IBD matches between admixed and reference populations (S22 Fig and S23 Fig). We find that consistent with the ASPCA, the most IBD sharing with European populations occurs between admixed populations and Iberian populations. The Peruvian individuals also share a much higher amount of IBD with the Andean Native Americans such as the Quechua and Aymara, as suggested by the ASPCA plots.

Taken together, the ASPCA and IBD results suggest that admixture between Europeans and Native Americans occurred in multiple locations throughout the colonization of the Americas and involved many different Native American populations. Individuals in our study were collected from major metropolitan regions in South American countries (Argentina, Peru, Chile, Colombia) or are recent US immigrants (Ecuador, Colombia). Our sample is thus an important reflection of the admixture patterns in cosmopolitan areas of these South American countries. While not fully representing the breadth of diversity across South America, the unique signatures of each of these populations gives deeper insight into regions that have historically been understudied. Important insights into population structure have been discovered using similar sampling schemes in previous work [6,18]. A more comprehensive sampling scheme throughout South American countries will help to reveal finer population structure patterns [17].

### Migration modeling from ancestry tracts

Analyzing the length of contiguous tracts of the same ancestry in an admixed population can help to determine the timing of admixture events and subsequent migrations. We used the program Tracts [37] to fit multiple models of admixture to the observed data. Tracts uses an optimization function to fit the ancestry tract length distribution under a given population history model to the observed tract length distribution. It also estimates the timing of major admixture and migration events within the model. Multiple models can be compared based upon their observed likelihoods given the data.

**Fig 4:**
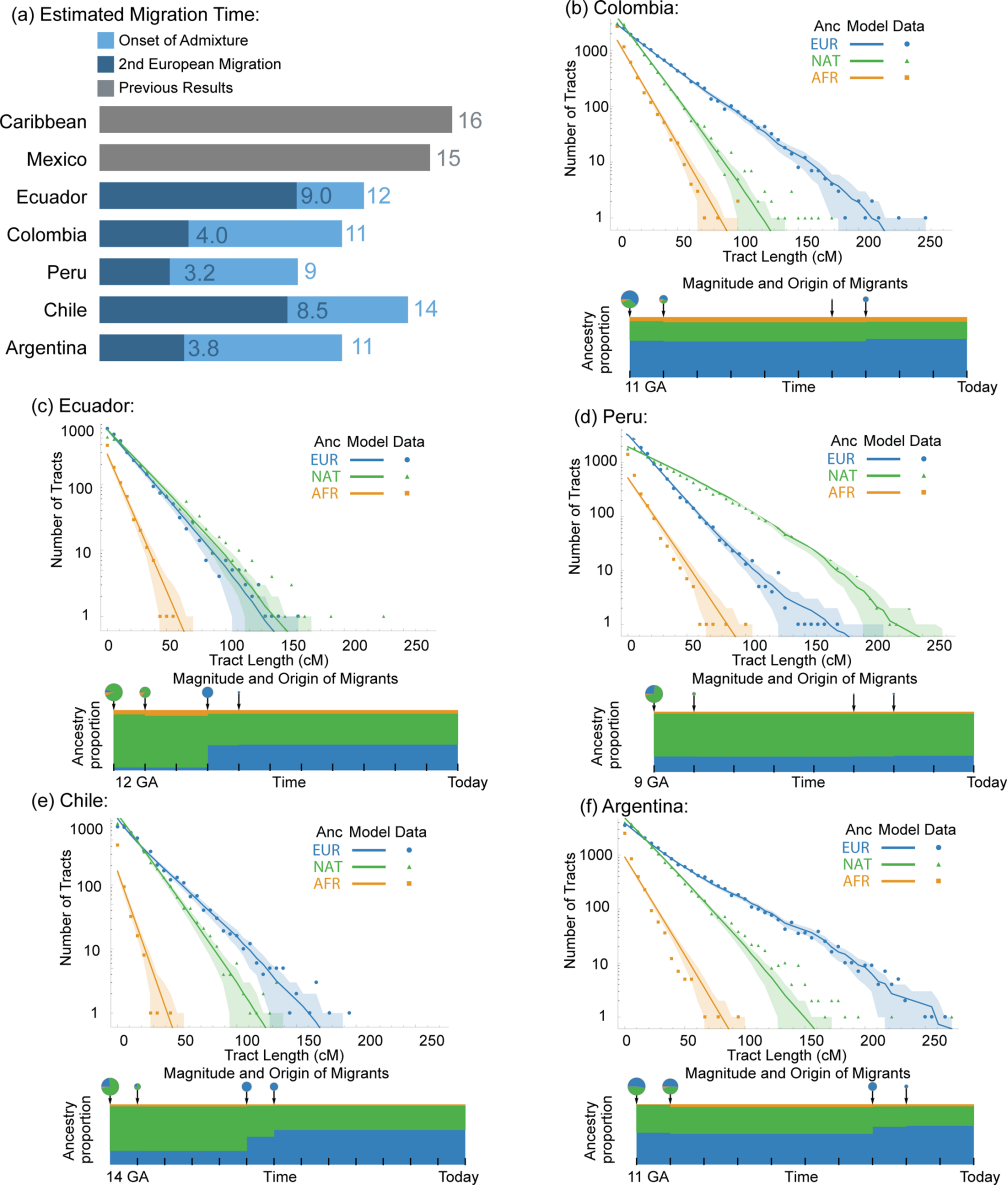
Tract length analysis and admixture times. (a) Bar graph comparing estimated migration times for the best-fitting model in each of the South American countries (blue) with previously published migration times for the Caribbean islands [6] and Mexico [40] (grey). The estimated second European migration time (in generations ago) is shown in dark blue for the models. (b-f) Best-fitting Tracts model for Colombia (b) Ecuador (c), Peru (d), Chile (e), and Argentina (f). These models have an initial admixture event between Native American and European populations followed by a pulse of African migration, subsequently followed by an additional European migration event. The ancestry tract decay (top) compares the expected and observed tract length distributions based upon the best-fit model, with the shaded area indicating the 68.7% confidence interval. The points indicate the observed values. The migration model (bottom of each graph) shows the change in admixture proportion over time. From left to right along the time scale in generations ago (GA), the circles on top indicate the occurrence of admixture events, with the size of the circle corresponding to the magnitude of incoming migrants from each of the ancestral populations.

To infer the genetic migration history of South American Latinos, we compared three potential hypotheses (S24 Fig). First, we fit a model with a single admixture event between Europeans and Native Americans followed by a migration of Africans. We considered this model our base model, reflecting the initial colonial-era contact followed by slave importation. Next, we fit a second model with an additional European migration event. A third model consisted of the base model with an additional African migration event. We compared the fits of the models using the observed likelihoods. The tracts model makes an assumption about independence of the tract length distributions that may not necessarily be true and can cause likelihood based methods to falsely reject a model in favor of a model with more ancestry pulses [38]. In order to ensure we were correctly rejecting the single pulse model, we compared our observed changes in likelihood to the changes in likelihood seen in 1000 simulations of the single pulse model (see Methods). We find that for all of the admixed populations studied, the model with the extra pulse of European migration was preferred (P < 0.001 for Argentina and Colombia, P = 0.002 for Peru, P = 0.003 for Ecuador, and P = 0.027 for Chile, S28 Fig). The best fitting models for each population are shown in Fig 4. This result suggests a demographic history of continued European migration to Latin South America. This is in contrast to many countries within the Caribbean, where Tracts models estimated multiple pulses of African migration [6]. This contrast reflects historical differences in the slave trade among regions of the Americas and its differential impact in present day Latino populations. While African slavery was extensive throughout colonial Spanish America, it is estimated that only 12.9% of the total number of slaves disembarked in colonial Spanish America [39]. Of the estimated 1.5 million slaves imported into Spanish colonies, over 80% of them disembarked in Central America, Cuba, and Puerto Rico [39]. While Rio de la Plata was a major slave destination, only about 83,000 of the estimated 1.5 million slaves sent to Spanish Colonial America disembarked at this port [39]. While disembarkation location is not necessarily indicative of a slave’s final destination, it gives a broad picture of the regional differences in the slave trade. Some of the difference in ancestry proportions and demographic models between the Caribbean and South America could be related to these regional differences.

A unique feature of South American migration history is the timing of European contact in colonial times. According to the best-fitting Tracts models, we can infer a rough estimate of the number of generations that have passed since the initial admixture and the subsequent migrations in each South American population. The estimates of these parameters are shown in S3 Table and in Fig 4 we have plotted the tract length distributions of the most likely models (as determined by the simulated likelihood comparison) for each South American population. The maximum-likelihood time of initial admixture between Native Americans and Europeans ranges from 9 to 14 generations ago among the studied populations, representing the youngest estimate in mainland Latin America. Previous studies of mestizo populations throughout South America have shown estimates of mean time to admixture between 6 and 14 generations [13,16], a range that our models agree with. The models return the maximum likelihood estimate for the time of onset of admixture in the entire population (once additional pulses have been taken into account). This, however, does not necessarily equate to an estimate for the earliest possible time that admixture may have taken place. The discrepancy between the recorded initial time of colonization and the onset of admixture described in our and previous work is likely due to many factors, including the fact that the admixture process occurred over time in the colonies and that further immigration occurred throughout the 16^th^, 17^th^, and 18^th^ centuries [22].

Our models argue for a more recent European migration into South America compared to that in the Caribbean and Mexico [6,40], consistent with the colonial history in the region. Strong pulses of European migration occurred between 3 and 9 generations ago in Colombia, Ecuador, Peru, Chile, and Argentina (Fig 4A). This more recent European migration into southern South American countries is consistent with historical records of European migration from Iberia and Southern Europe throughout the 19^th^ and 20^th^ centuries [22]. For example, it is estimated that over 4.7 million individuals immigrated into Argentina between 1880 and 1930 [24], and that the majority of these immigrants were of European origin. Our models suggest a strong pulse of European immigration between 3 and 4 generations ago in Argentina, which is consistent with the recorded history.

The Tracts model for Argentina (Fig 4), however, is not able to properly fit the tail of the distribution, where longer Native American tracts were observed. This is likely due to the high variance in ancestry proportions in Argentina. Because of this, the admixture time estimates for individuals in Argentina may be strongly dependent upon subpopulation. This result argues for the need to study these populations at an even finer scale to help discriminate the complex local patterns of ancestry.

### Methodological considerations

Previous work has performed ASPCA ancestry analyses using both trio [6] and population [5] phased data. Here, we show that the results of these analyses between trio-phasing and pop-phasing samples are similar. Trio phasing generally produces more accurate haplotypes than population phasing. This could affect the results of ancestry deconvolution methods that rely upon long range phasing information, such as ASPCA and Tracts. However, we find no significant difference between trio and population phasing results when using RFMix’s phase correction feature. In the paper describing the RFMix algorithm [41], Maples et. al. demonstrate that the RFMix phase correction feature produces highly accurate long range haplotypes in admixed populations even when population phasing was performed. To assess the differences between trio-phased and population phased data, we compared ASPCA and Tracts results from the 1000 Genomes Peruvian and Colombian individuals between the different phasing approaches. For ASPCA, we find that the population and trio-based methods return similar results for both the Native American and European ancestry (S31 Fig). To assess the effect of phasing on the IBD analysis, we compared the results of IBD tract length analysis of trio-phased and pop-phased samples. The trio-phased IBD analysis finds an increased number of IBD tracts, however, the proportion of European, Native American, and African tracts is very similar (S32 Fig) and the length distribution of the tracts is similar. We find the IBD tracts of each population have a Spearman correlation of 0.995 for the European tracts, 0.996 for the Native American IBD tracts, and 0.952 for African IBD tracts. Thus, we find no evidence of systematic bias in the IBD analysis due to the population phasing. For Tracts analysis, the trio-phased Tracts result has an earlier onset of admixture than the population-phased samples in the both populations. Specifically, we estimate 10 generations to the onset of admixture in the population-phased 1000 Genomes Peruvians vs. 9 generations for the same individuals when trio-phased. For the 1000 Genomes Colombians, we estimate 12 generations for both the population-phased and trio-phased data. Therefore, the admixture onset times calculated here may be slightly biased towards overestimating the initial onset of admixture. These tests indicate that using population-phased samples in combination with RFMix’s phase correction abilities in our ancestry analysis pipeline introduces little bias to the results.

### Conclusion

South America has experienced major demographic shifts caused by multiple Native American migration routes into the region, European colonization and the African slave trade and, more recently, by continued inter-continental and local migration. A number of genetic differences distinguish these populations from other Latino groups. A highly structured pre-Columbian population transmitted local patterns of variation that cluster by country and are not observed outside South America. The Native American ancestral component differs significantly throughout the populations in South America, with an especially striking difference between Andean and non-Andean populations. The later contact of Europeans and recent migrations from Southern Europe translates into a different European gene pool contributing to South American admixture compared to that involved in the initial pulses of migration into the Americas, particularly in Chile and Argentina. In particular, strong pulses of European migration identified in Argentina correspond to historical records of strong Southern European immigration to the region. These findings not only shed light on the reconstruction of demographic events associated with population admixture in South America, but also on population-specific genetic profiles defining particular Latino groups, which have important implications for the expected relative proportion of deleterious mutations that can be detected via association studies in the region. The extensive structure observed in subcontinental ancestry between different populations also suggests that medically relevant genetic variation may vary between populations, demonstrating the need to ensure representation of diverse populations in future genetic association studies.

## Materials and Methods

### Ethics statement

Newly collected samples were obtained with individual written consents provided by voluntary participants recruited under IRB approval of the Oklahoma Medical Research Foundation (number 09–23), and the local IRB of each participating institution at recruitment sites in South America.

### Data collection and sampling

The populations included in this study combine newly collected samples and publicly available data from relevant samples. Participants from Argentina, Peru, and Chile were recruited as part of a larger study aimed at understanding the genetic basis of Lupus in Latinos [42]. The complete GWAS cohort was genotyped using Illumina OMNI1 arrays and only healthy controls from the three aforementioned countries of origin were considered for inclusion in the present population study (n = 266). Markers with less than 99% call rate were filtered and a total of 694,834 SNPs remained for intersection with additional data sets. Individuals from Argentina, Peru, and Chile were recruited at hospitals within major metropolitan regions of each country, including Lima (Peru), Santiago (Chile), Cordoba (Argentina), Mar del Plata (Argentina), Rosario (Argentina), Santa Fe (Argentina), Mendoza (Argentina), and multiple sites in Buenos Aires (Argentina). Most individuals were sampled from public hospitals. Only 6 of the 175 individuals from Argentina were sampled from private hospitals. The number of individuals sampled from each hospital is reported in S5 Table. Individual genotype data for the 266 newly genotyped individuals will be made available through dbGaP under the Susceptibility Genes for SLE of Amerindian Origin in Hispanics study. Individuals from Ecuador and Colombia were sampled in the New York City area as described previously in Bryc et al [18] and were genotyped using Illumina 650K SNP arrays and filtered as described therein. We also included genotype data from unrelated Peruvian and Colombia individuals from the 1000 Genomes Project, who were sampled in Lima (Peru) and Medellin (Colombia). We then removed admixed individuals with an estimated PLINK kinship score of greater than 0.25.The final data set of unrelated admixed individuals consisted of 175 Argentinian samples, 119 Peruvian samples, 27 Chilean samples, 19 Ecuadorian samples, and 96 Colombian samples. Different intersections between data sets resulted in varying SNP densities for each of the analyses as described below and are summarized in S4 Table. Statistical analysis and plotting were performed in R version 3.1.2 [43] and using ggplot2 [44].

### Admixture and PCA analysis

We performed global ancestry analysis by combing the admixed South American individuals with reference panels representing each continent. For West Africa, we used genotypes from 50 Yoruba individuals in 1000 Genomes [3]. For Asia, we used 50 of the Han Chinese from Beijing (CHB) individuals. A large proportion of the admixed ancestry for the South American individuals was expected to be from European and Native American populations of diverse origin. Therefore, we used larger panels for these groups in the global ancestry analysis. For European populations, we used a subset of 204 individuals from the POPRES sample that capture the North-South gradient of genetic diversity [1]. For Native American samples in the global analysis, we used the individuals previously genotyped by Reich *et al*. [9]. This data set comprises 49 Native American populations from throughout the Americas with genotype data available for 364,470 SNPs. The combined data set had a total intersection of 24,592 SNPs. For most of the analyses described, we have grouped these Native American populations by sampling location and thus will refer to them as “Northern Amerindian”, “Central Amerindian”, “Southern Amerindian”, and “Andean Amerindian” (S1 Table). Principal Components Analysis (PCA) was performed on the combined dataset using the EIGENSOFT method implemented in the Plink software package [45,46]. We used the unmasked version of the Reich *et al*. data set, since standard PCA is highly sensitive to missing data. We next performed ADMIXTURE [25] analysis on the combined data set. However, some of the individuals in the Reich *et al*. data set have significant European ancestry, and this is reflected in the global PCA results. ADMIXTURE is less affected by this additional European admixture component or by missing data in the Native American samples than PCA. We performed ADMIXTURE with the unmasked Native American samples. ADMIXTURE models were explored at varying number of *K* clusters from *K* = 2 through *K* = 15. We observed the lowest CV error at K = 13. Higher order *K*s resulted in within-population clusters rather than population-level signals and were thus not considered. To compare global ancestry results to previous analyses, we combined our data with previously published individuals from the Caribbean [6] and performed ADMIXTURE at K = 4. We compared values of African ancestry using the Wilcoxon signed-rank test. To assess sex-biased ancestry in admixed female individuals, we combined our data with POPRES Europeans, 1000 Genomes Africans, and Native Americans from South America [6,47]. After excluding admixed males from the analysis, we had admixed individuals from only four populations: Argentina, Chile, Colombia, and Peru. We compared the ADMIXTURE estimates at K = 3 of X-chromosomal and autosomal ancestry from admixed females.

### Phasing and local ancestry inference

For local ancestry analysis, we used continental reference panels to identify each of the three continental-level ancestry components along each admixed chromosome. To maximize phasing accuracy, we chose representative populations for which trio samples were publicly available. To represent Africa, we used the YRI population from 1000 Genomes [3]. For Europe, we used the CEU population from the 1000 Genomes Project. For Native American ancestry, we used a combined set of Maya and Tepehuano individuals from Mexico [5]. In previous studies we have demonstrated that for continental-level inferences, the population chosen as reference plays a minor role in the accurate identification of highly diverged components of African, European and Native American ancestry [6,36,40]. Phasing was performed independently on each of the three reference panels and on the admixed individuals using SHAPEIT [48] with default parameter settings.

From the phased data, we used a discriminative modeling approach implemented in RFMix to perform local ancestry inference [41]. RFMix is a local ancestry inference method that uses random forests to infer the local ancestry of chromosomal segments. Input to RFMix consists of phased genotype data from a set of reference panels for each population and phased genotype data from admixed individuals. Using the genotype data from the reference panels, RFMix builds a local ancestry model for each of the phased admixed chromosomes by training a random forest classifier. It also performs phase correction to improve the accuracy of haplotypes in the admixed individuals. Finally, RFMix uses an EM algorithm to iteratively improve local ancestry calls. We ran RFMix on our data set with the phase correction feature enabled and performed two rounds of the EM algorithm to improve local ancestry calls. We used the population phased version of the RFMix program, which assumes population phasing for the admixed individuals. We used the default window size of 0.2 cM, the default number of trees (100) and the default RFMix model of admixture occurring 8 generations previously. We used the three continental reference panels (African, European, and Native American) in RFMix to identify the genomic regions in admixed individuals that are likely to have originated from each continent. RFMix generated a local ancestry call at each site for each haploid genome.

### Ancestry-specific PCA analysis

We used the program PCAmask to perform ancestry specific PCA analysis (ASPCA) [6]. The input to the program consists of admixed individuals with local ancestry calls and a subcontinental reference panel of the ancestry under investigation. PCAmask masks the loci in the haplotypes of the admixed individuals that have local ancestry that does not correspond to the given continental population. Because of the high amount of missing data this masking generates, PCAmask uses a modified version of subspace PCA [49] to implement an ancestry specific PCA. The analysis combines both the admixed individuals and the subcontinental ancestral population into the same PCA space for analysis.

To run ASPCA on the European component of the admixed individuals ancestry, we combined our admixed individuals with the POPRES European data set [1]. Because both of our Latino data sets were genotyped using different Illumina SNP arrays, and the POPRES European reference panel [1] was genotyped in a different platform (Affymetrix 500K), the intersection of all three data sets would lead to a substantially reduced marker overlap. Therefore, we performed the ASPCA analysis of the European component separately for the individuals from Ecuador and Colombia (genotyped on Illumina 650K arrays) and the individuals from Peru, Chile, and Argentina (genotyped on Illumina OMNI1 arrays). The intersection of the POPRES samples and the Colombians and Ecuadorians contained 21,570 SNP markers while the intersection of POPRES and the Argentinian, Peruvian, and Chilean samples contained 35,070 SNPs. The intersection of the three data sets contained too few SNPs for analysis. To further assess the European ancestry of the Colombians and Ecuadorians, we combined these individuals with previously published genotype data from Canary Islanders [50]. We used SHAPEIT and RFMix as above to mask the Canary Islanders using a European and a North African reference panel [51]. We performed ASPCA analysis with the POPRES individuals, Canary Islanders, Colombians, and Ecuadorians. We masked all non-European regions of the genomes of the Canary Islanders, Colombians and Ecuadorians.

For the ASPCA analysis of the Native American component, we used a combined data set including the admixed Latino individuals from all 5 South American countries investigated and the unmasked Native American reference panel from Reich *et al*. [9]. We excluded admixed individuals with less than 25% Native American ancestry inferred through local ancestry inference. In addition to masking regions of the admixed genomes that corresponded to non-Native American ancestry, we also masked loci where RFMix reported a less than 95% posterior probability of the inferred ancestry. After these filters, we considered 182 Argentinian, 236 Peruvian, 51 Chilean, 114 Colombian, and 38 Ecuadorian haploid genomes for analysis. The final intersection of these data sets contained 142,161 polymorphic sites. The first comparison with the Native American reference panel identified many of the extremely homogenous Native American groups as outliers (S7 Fig). This included the Brazilian Surui and the Costa Rican Cabecar individuals, among other populations. We also removed North American, Na-Dene and Aleut groups from downstream analyses, as they are geographically distant from South America and further analyses indicate these individuals were unlikely to be involved in South American Latino admixture. After excluding these individuals, we re-ran ASPCA and found that there was a strong gradient of dispersion within the Native American reference panel (S8 Fig). This gradient correlated strongly with ADMIXTURE estimated European ancestry components in linear regression (p < 2 x 10^−16^ for ASPC1 and ASPC2, S9 Fig and S10 Fig). We hypothesize, therefore, that this gradient is an artifact of recent European admixture in Native American populations. We therefore excluded any individuals from the Native American reference panel with greater than 10% European ancestry. After this filter, we re-ran ASPCA. This data set included 108 Andean, 132 Central Native American, 118 Northern Native American, 122 Southern Native American haplotypes. A table of the specific numbers of haplotypes from each Native American population is provided in the Supplement (S2 Table). ASPCA was performed with this reduced Native American reference panel of 480 haploid genomes in combination with the 182 Argentinian, 236 Peruvian, 51 Chilean, 114 Colombian, and 38 Ecuadorian masked haploid genomes.

To further investigate the relationships between admixed individuals throughout South America, we performed an identity by descent analysis using the program GERMLINE [35]. For the IBD analyses, we used the default settings of GERMLINE, with settings of bits = 128 and allowing a maximum of two homozygous marker mismatches per IBD slice (-err_hom) and a maximum of 0 heterozygous marker mismatches per IBD slice (-err_het). For IBD analyses involving combining the IBD tract information with local ancestry assignments, we used the haploid mode of GERMLINE to look for IBD matches between individual haplotypes inferred through Shapeit and RFMix. The haploid mode of GERMLINE is more conservative than the diploid mode, as a phasing switch error will interrupt an IBD match. The minimum length for IBD segment detection was 3 cM. For IBD within admixed individuals, we used the rephased alleles output from RFMix. We then compared the locations of IBD matches to the inferred local ancestry calls throughout each IBD tract. To better understand the IBD relationships between admixed populations and subcontinental reference populations, we compared estimated IBD tract lengths between our admixed individuals, the Native American ASPCA subset of the Reich et. al. data set, and the European 1000 Genomes populations. The European 1000 Genomes individuals were used in place of the POPRES data set for IBD detection due to the low marker overlap with the POPRES individuals. We calculated the sum of the total length of IBD sharing between the admixed populations and reference populations. This value was normalized by the product of the numbers of individuals in each respective admixed and reference population. Standard error was calculated using chromosome weighted jackknife sampling [52].

### Tract length analysis

To infer migration times and admixture events, we used the program Tracts [37]. Tracts fits a migration model to the local ancestry tract length distribution within a population. In doing so, it also infers the number of generations since the admixture events in each model. We ran the Tracts program on multiple demographic models for each of the countries in South America. We excluded individuals with estimated ancestry proportions of greater than 95% of one continental population (European, African, or Native American) as these individuals represent either non-admixed individuals or extremely recent migrants. After this filter, we had a total of 154 Argentinians, 117 Peruvians, 95 Colombians, 27 Chileans, and 19 Ecuadorians included in the Tracts analysis. We compared the likelihoods of each ancestry model. The Tracts model makes an assumption about the independence of the distribution of ancestry tracts that may not necessarily true, this is discussed further in Liang and Nielsen [38]. This assumption may skew the reported likelihood of the model, causing the model to, in certain situations, be biased towards favoring the inclusion of additional ancestry pulses. To ensure our results were not affected by this bias, we used forward simulation to generate a tract length distribution given the estimated parameters from the single pulse model for each population. We then for the simulated data calculate the change in likelihood between the single pulse and two pulse models. We compare our observed change in likelihood against 1000 iterations of the simulated changes in likelihood when the true model is the single pulse model. If our observed likelihood change is of greater magnitude than 95% of the simulated likelihood changes, we rejected the model with the single pulse of migration.

We tested three models (S24 Fig) based upon our hypotheses for the recent history of South America. The first model was a base model with two parameters and included a single admixture event between Native American and European populations followed by a pulse of migration of African individuals. The parameters for this model were time of original admixture and time of African migration. The second model had an additional pulse of European migration, while the third model had an additional pulse of African migration. Each of these two models had two additional parameters, one corresponding to the magnitude and one corresponding to the time of the subsequent migration.

### Assessing differences between trio and population phasing

In order to assess the possible effects of using population-phased samples instead of trio-phased samples in this analysis, we compared results from the 1000 Genomes PEL and CLM cohorts. We generated one set of haplotypes and local ancestry calls through population phasing using SHAPEIT including only the parents. We generated another set of haplotypes and local ancestry calls by performing a trio-based phasing through SHAPEIT with the PEL and CLM analysis. We performed a comparison of the ASPCA, IBD, and Tracts analysis between these two data sets. For the ASPCA analysis, we used the same reference panels of Native Americans and Europeans as in previous analyses. We compared the correlation between tract lengths of different ancestries in the haploid version of the GERMLINE IBD program. We compared the estimated migration times between the trio-phased and pop-phased ancestry tract length analysis.

### The combinations of different data sets used in this manuscript

Throughout the manuscript, multiple combinations of admixed individuals and data sets are used. This is due to the different requirements of each of the analyses in terms of SNP densities and reference populations. The major difference is that admixed individuals, continental reference panels, and the Native American reference panel were genotyped on various Illumina genotyping platforms while the POPRES data set was genotyped on the Affymetrix GeneChip 500k. For local ancestry inference, the combination of admixed individuals and reference panels contained approximately 190,000 SNPs. These data sets were used for the local ancestry estimation and tract length analysis. For combinations of the admixed individuals and the Reich et. al. data set of Native Americans, the average SNP density was approximately 140,000. These include the Native American ASPCA and IBD analysis. For combinations with the POPRES data set, which was genotyped on the Affymetrix GeneChip 500k, combined data sets had a much lower SNP density, approximately 30,000 SNPs. These data sets were used in the global admixture analysis and European ASPCA. The data sets used for each of the analyses are summarized in S4 Table.

## Supporting Information

S1 Fig Principal Components Analysis PC3 and PC4 of admixed and reference individuals. (PDF)

S2 Fig CV error for each ADMIXTURE K value. (PDF)

S3 Fig ADMIXTURE ancestry proportions at K = 4. (PDF)

S4 Fig ADMIXTURE proportions at K = 2 through K = 15. (TIF)

S5 Fig ADMIXTURE proportions at K = 2 through K = 15 with detailed labels. (TIF)

S6 Fig South American vs. Caribbean African ancestry. Boxplots are comparing ADMIXTURE estimates of African ancestry in the Caribbean Island individuals for Moreno-Estrada et. al. 2013 to African ancestry estimates in the South American individuals.
(PDF)

S7 Fig Autosomal vs. X-chromosomal ancestry. K = 3 ADMIXTURE proportions for admixed female individuals combined with a European, African, and Native American reference panel on autosomal chromosomes and on X-chromosomal markers.
(PDF)

S8 Fig Differences in X-chromosomal and autosomal Native American ancestry proportions by populations. (PDF)

S9 Fig European ASPCA with Canary Islanders. European ASPCA analysis of masked Colombians, Ecuadorians, Canary Islanders and the subcontinental POPRES reference.
(PDF)

S10 Fig Native American ASPCA analysis with full reference panel. Admixed individuals are in purple while Native American individuals are in greens and reds. Outlier populations (see outlying clusters on PC2) and Eskimo-Aleut and Na-Dene populations were removed from the analysis.
(PDF)

S11 Fig Native American ASPCA after removing outlier groups from the analysis. Admixed individuals are in purple and Native American individuals are in greens. The spread towards the bottom left corner is due to European admixture (see S8 and S9 Fig).
(PDF)

S12 Fig European ancestry proportion vs. ASPC1. Proportion of European ancestry (estimated through ADMIXTURE at K = 4) in unmasked Native American Samples vs. position on ASPC1 in S7 Fig.
(PDF)

S13 Fig European ancestry proportion vs. ASPC2. Proportion of European ancestry (estimated through ADMIXTURE at K = 4) in unmasked Native American Samples vs. position on ASPC2 in S7 Fig.
(PDF)

S14 Fig Sampling city latitude vs. ASPC1 for Argentinians. Colors correspond to the sampling city.
(PDF)

S15 Fig Sampling city latitude vs. ASPC2 for Argentinians. Colors correspond to the sampling city.
(PDF)

S16 Fig Sampling city longitude vs. ASPC1 for Argentinian individuals. Colors correspond to the sampling city.
(PDF)

S17 Fig Sampling city longitude vs. ASPC2 for Argentinian individuals. Colors correspond to the sampling city.
(PDF)

S18 Fig IBD tracts. Histogram of IBD tracts shared within and between populations by the most common ancestry within each IBD tract.
(PDF)

S19 Fig Shared IBD tracts. Comparison of the proportion of European ancestry in an IBD tract compared with IBD tract length for tracts shared between populations.
(PDF)

S20 Fig Within population IBD tracts. Comparison of the proportion of European ancestry in an IBD tract compared with IBD tract length for tracts shared within populations.
(PDF)

S21 Fig Population specific IBD tracts. Comparison of the proportion of European ancestry in an IBD tract compared with IBD tract length for tracts shared within each individual population.
(PDF)

S22 Fig IBD sharing with European populations. Normalized length of IBD matches between admixed populations and European populations from 1000 Genomes. For each population, the calculated value and jackknife standard error bars are shown.
(PDF)

S23 Fig IBD sharing with Native American populations. Normalized length of IBD matches between admixed populations and Native American populations. For each population, the calculated value and jackknife standard error bars are shown.
(PDF)

S24 Fig Tracts model schematic. Schematic of the three models tested in Tracts. The top represents the base model, the second panel represents the models with an additional pulse of European migration, and the third panel represents the model with an additional pulse of African migration.
(PDF)

S25 Fig Tracts base model. Tracts Analysis results for the base model, which has a European/Native American admixture event followed by a pulse of African migration. Results are shown for each of the countries. The first half of each panel plots the distribution of ancestry tract lengths found within the individuals. The line plots the expected distribution given the fitted Tracts models, with the shading indicating the 68% confidence interval. The second plot on each panel shows the change in ancestry proportions over time within the admixed populations. Tracts of specific ancestries are colored as follows: blue = European, green = Native American, orange = African.
(PDF)

S26 Fig Tracts European model. Tracts Analysis results for the Tracts model with an additional pulse of European ancestry. Results are shown for each of the countries. The first half of each panel plots the distribution of ancestry tract lengths found within the individuals. The line plots the expected distribution given the fitted Tracts models, with the shading indicating the 68% confidence interval. The second plot on each panel shows the change in ancestry proportions over time within the admixed populations. Tracts of specific ancestries are colored as follows: blue = European, green = Native American, orange = African.
(PDF)

S27 Fig Tracts African model. Tracts Analysis results for the Tracts model with an additional pulse of African ancestry. Results are shown for each of the countries. The first half of each panel plots the distribution of ancestry tract lengths found within the individuals. The line plots the expected distribution given the fitted Tracts models, with the shading indicating the 68% confidence interval. The second plot on each panel shows the change in ancestry proportions over time within the admixed populations. Tracts of specific ancestries are colored as follows: blue = European, green = Native American, orange = African.
(PDF)

S28 Fig Distributions of simulated likelihood changes between one and two-pulse tracts models. Here we have plotted the simulated likelihood differences between a single pulse and double pulse model of admixture for each population. The dashed red line indicates the observed likelihood change for each population. Note that for Argentina, the observed likelihood change (+220.1) was so great that it is not plotted within the axis limits. We simulated 1000 tract length distributions for each population based upon the best fitting single pulse model. We then assessed the increase in likelihood in a simulated environment that occurred with the addition of an extra pulse of European migration. If our observed likelihood change was greater than 95% of the simulated likelihood changes, we preferred the model with the extra European migration pulse.
(PDF)

S29 Fig PC1 vs. PC2 with population centroids and regions. Population centroids and shaded regions are plotted for all of the populations included in the analysis.
(PDF)

S30 Fig Native American ASPCA with population centroids and regions. Population centroids and shaded regions are plotted for the admixed populations in the Native American ASPCA analysis.
(PDF)

S31 Fig Comparison of ASPCA with population and trio-phasing in PEL and CLM individuals. (PDF)

S32 Fig Comparison of IBD Tract distributions between population and trio-phased PEL and CLM individuals. Data are shown on a log-scale to facilitate comparisons.
(PDF)

S1 Table Native American population names. Native American population names and geographic grouping. Also contains the language groupings found in Reich et. al. 2012 [9].
(XLSX)

S2 Table Haplotypes in ASPCA. Number of haplotypes from each Native American population included in the Native American ASPCA plot.
(XLSX)

S3 Table Tracts likelihoods. Likelihoods, BIC, simulated p-values, and migration parameters for each of the Tracts models tested.
(XLSX)

S4 Table Data sets used. Table of the data sets, number of SNPs, and number of individuals used for each of the analyses.
(XLSX)

S5 Table Sampling hospitals of admixed individuals. (XLSX)
