# Supplementary figures and images for: Genomic Insights into the Ancestry and Demographic History of South America
Source: PLoS Genet. 2015 Dec 4;11(12):e1005602. doi: 10.1371/journal.pgen.1005602 (PMC4670080; doi:10.1371/journal.pgen.1005602)

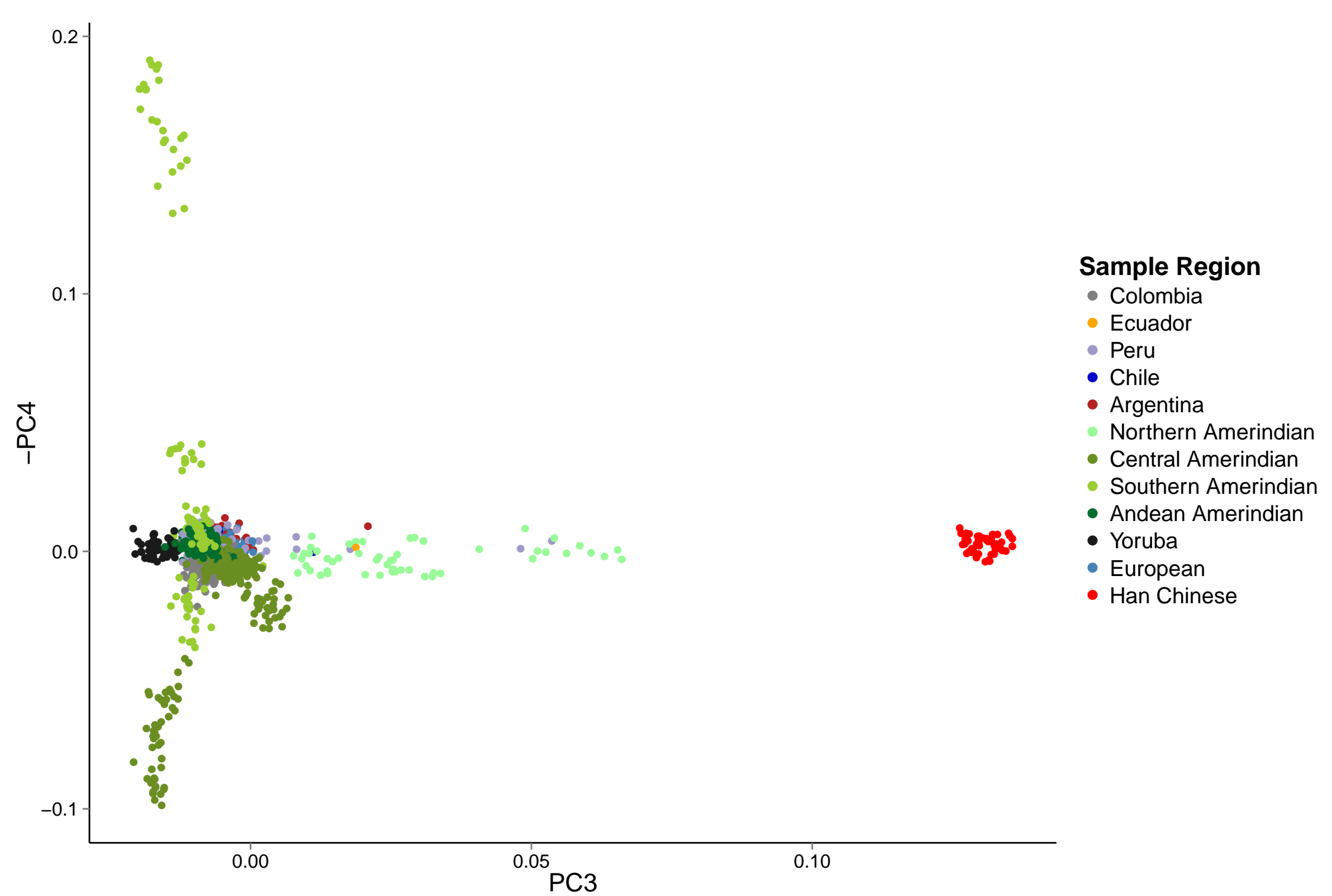

Supplement: S1 Fig — (PDF) [file pgen.1005602.s001.pdf]

Admixture CV Error

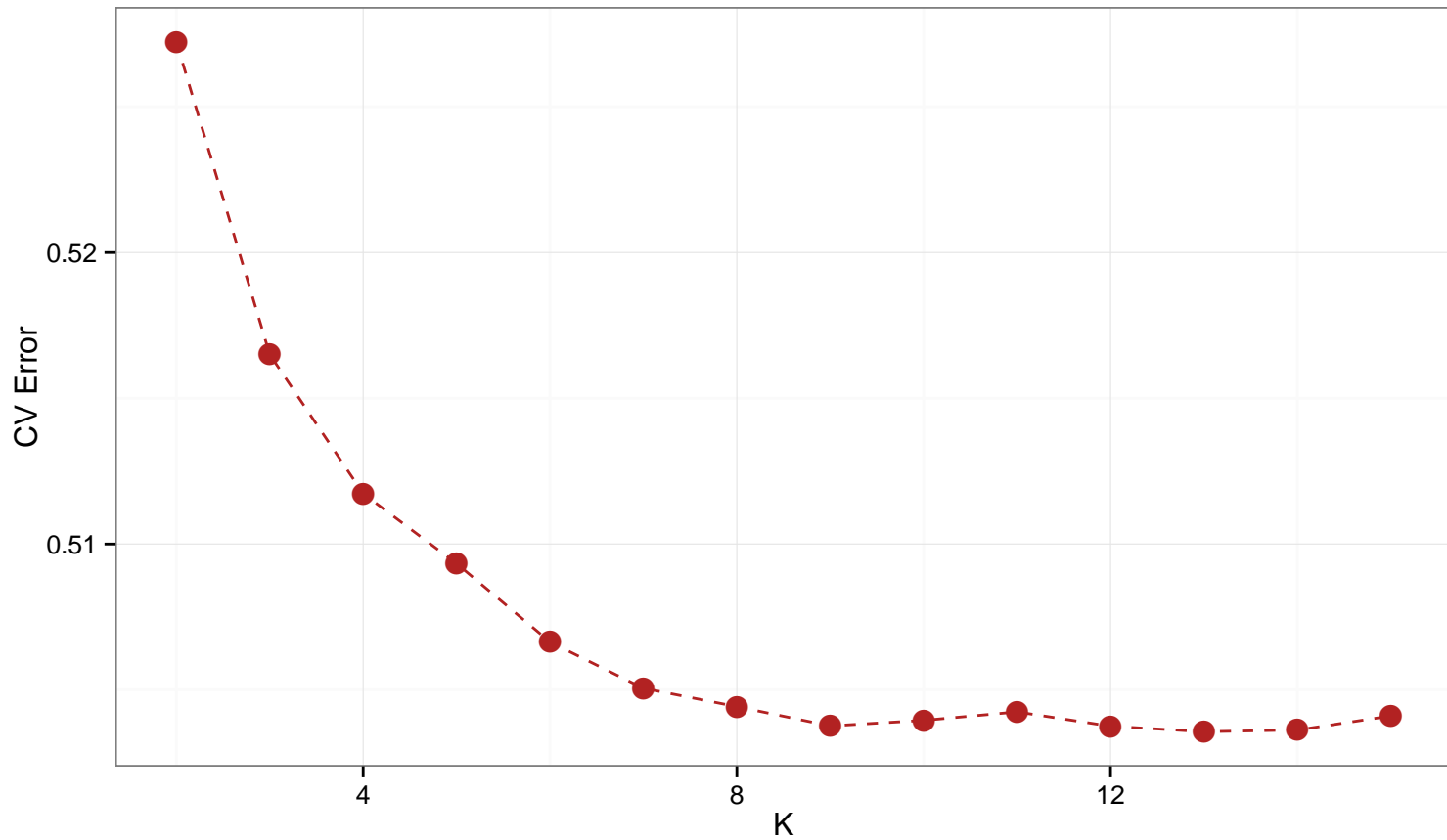

Supplement: S2 Fig — (PDF) [file pgen.1005602.s002.pdf]

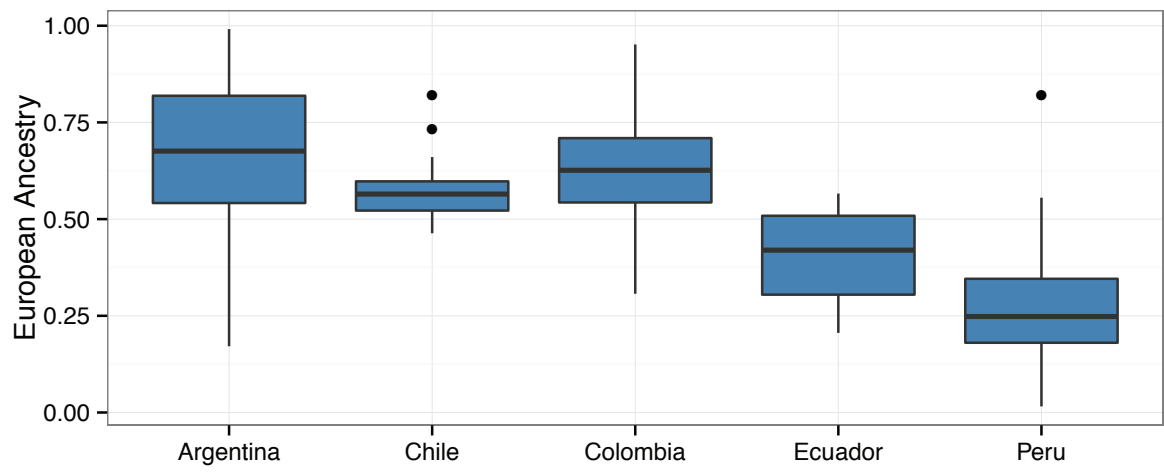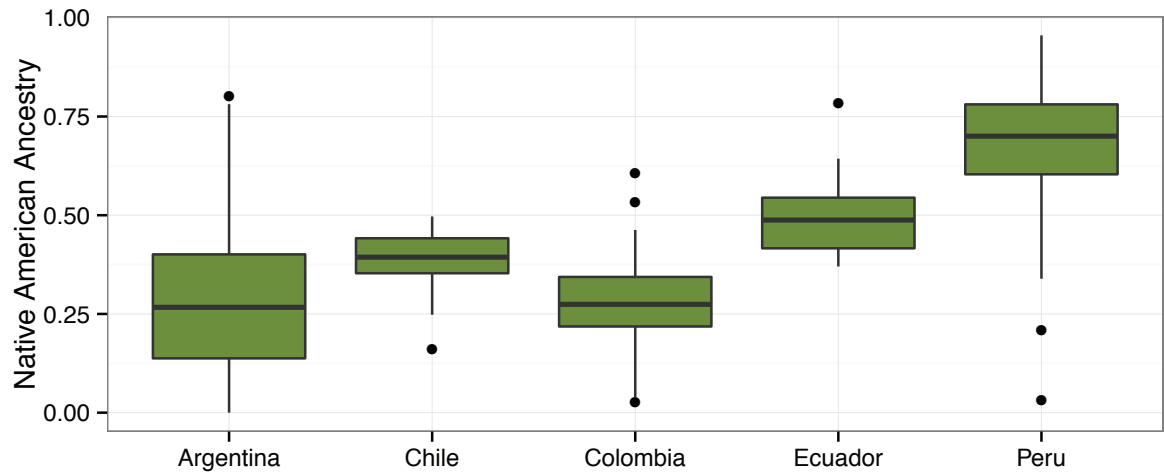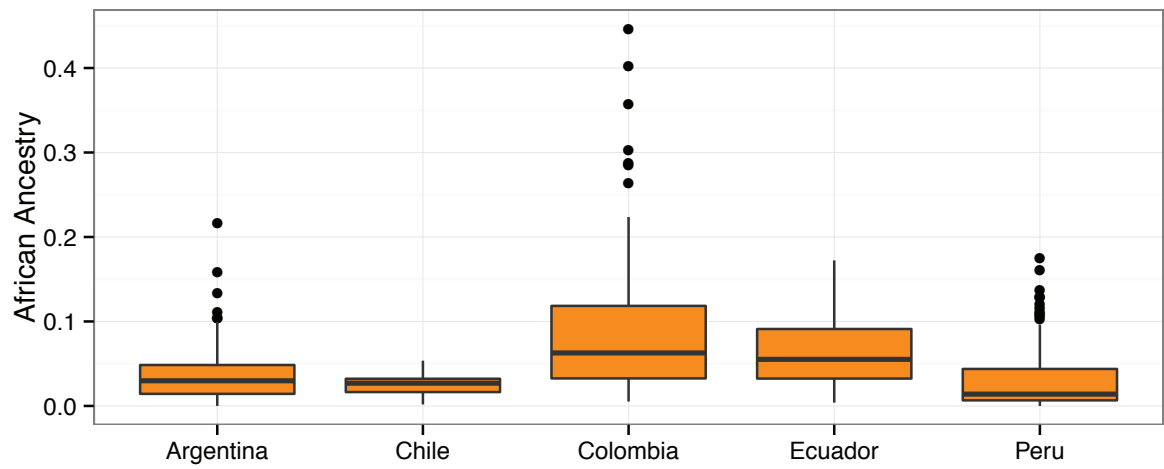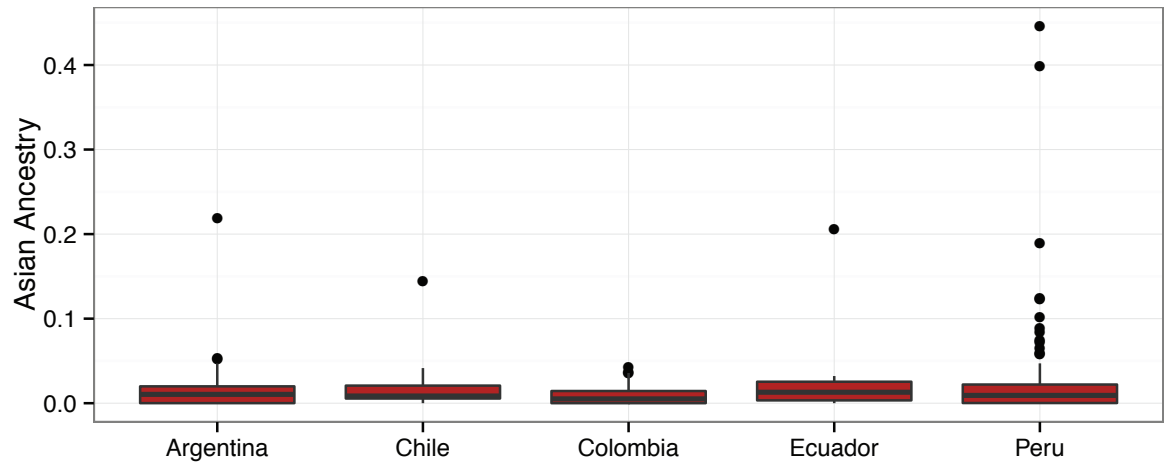

Supplement: S3 Fig — (PDF) [file pgen.1005602.s003.pdf]

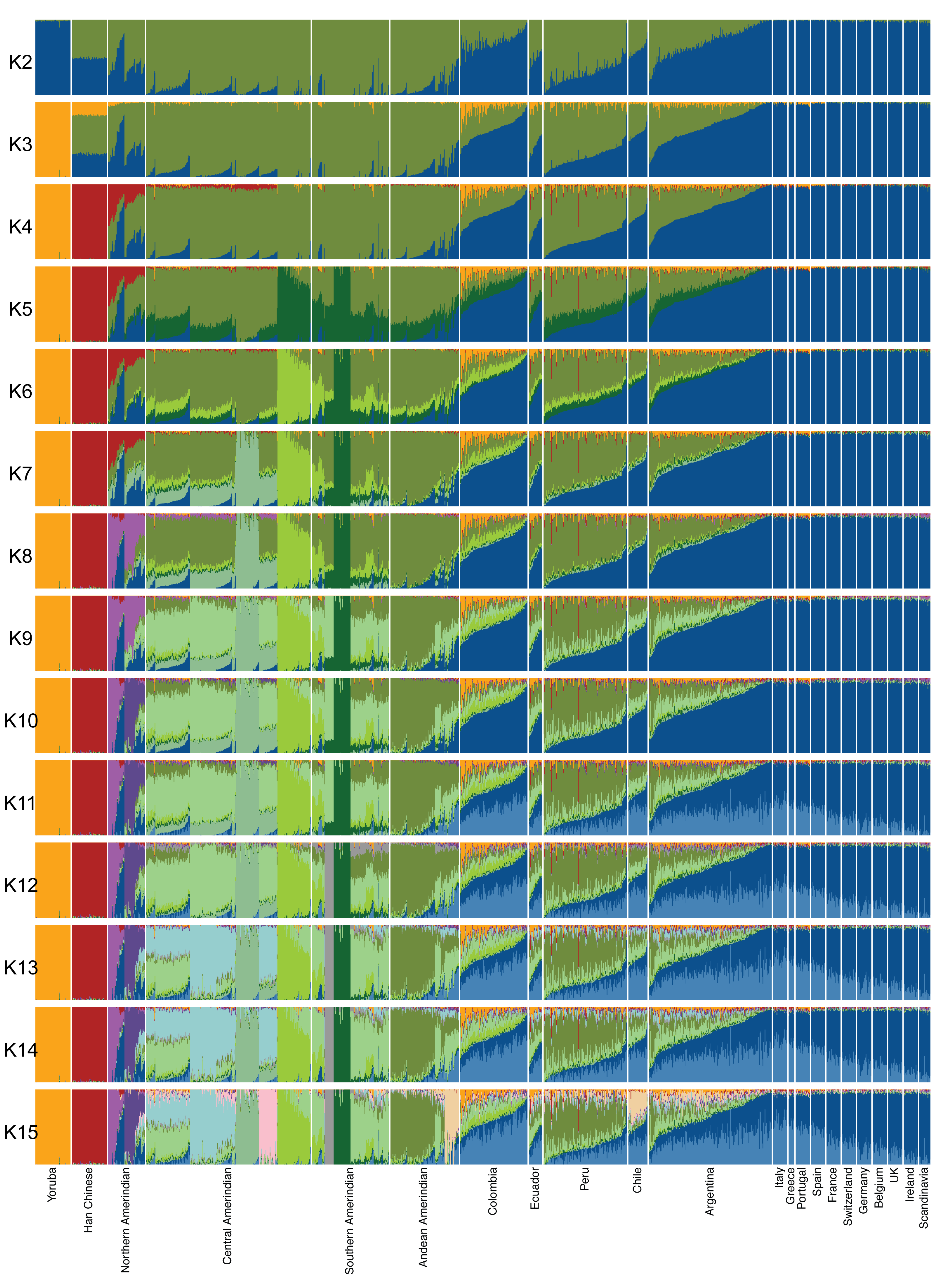

Supplement: S4 Fig — (TIF) [file pgen.1005602.s004.tif]

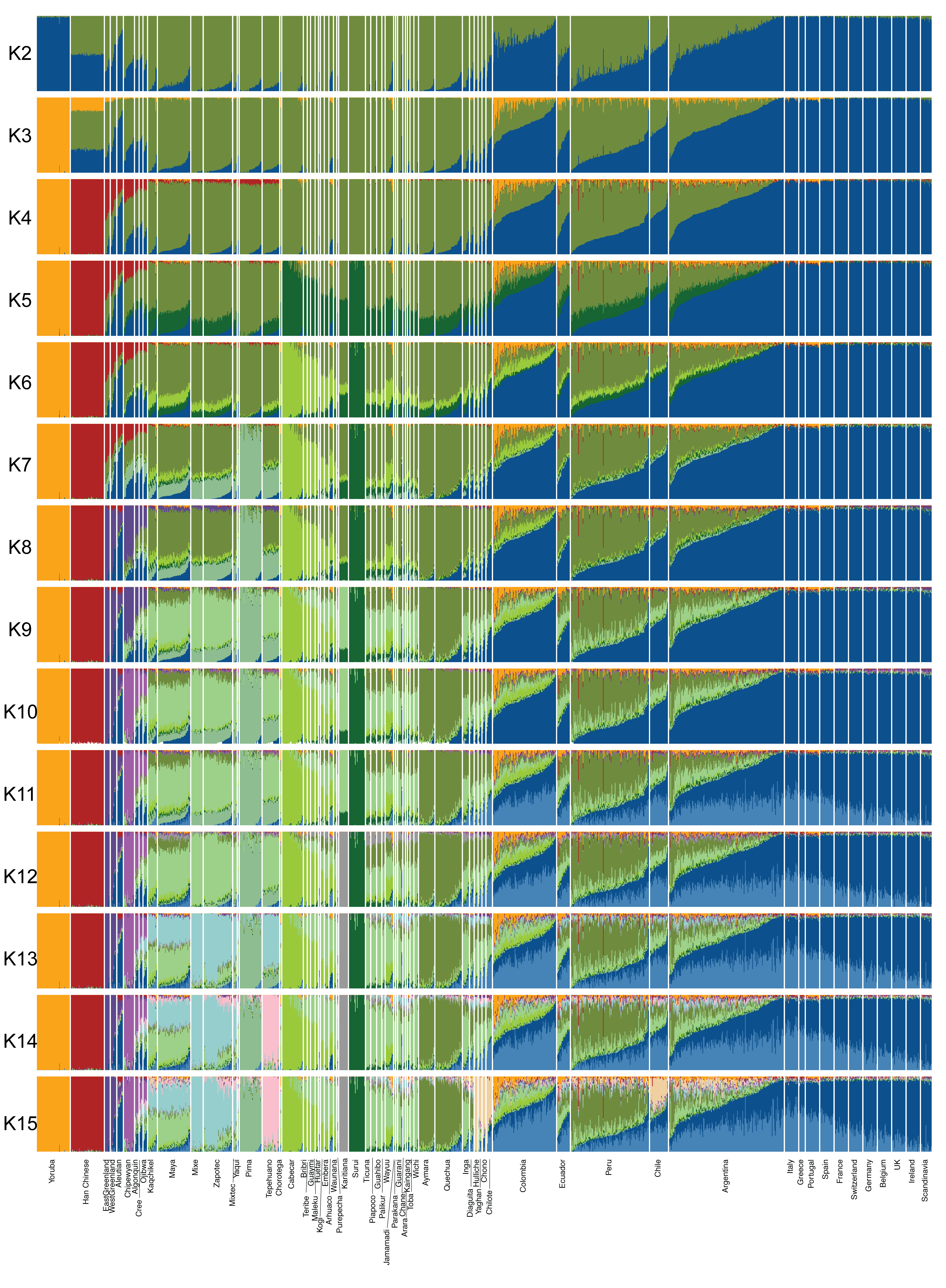

Supplement: S5 Fig — (TIF) [file pgen.1005602.s005.tif]

# African Ancestry in South America vs. Caribbean

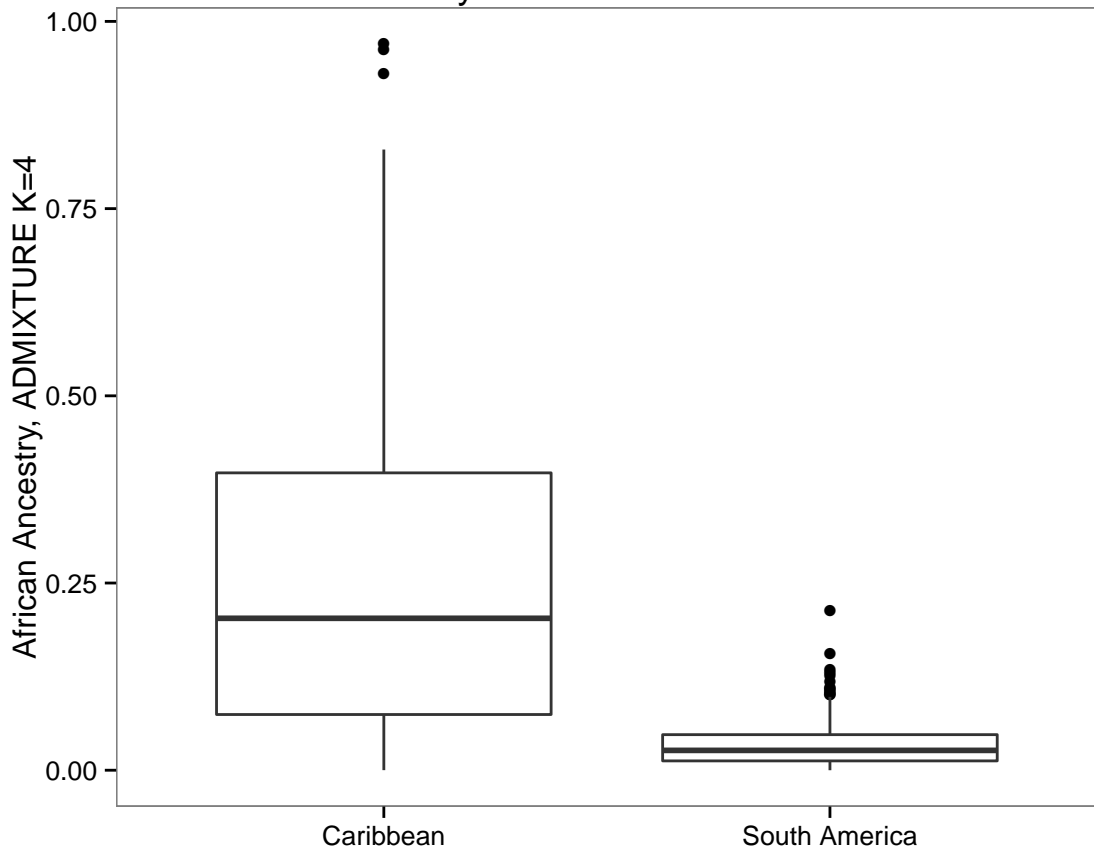

Supplement: S6 Fig — Boxplots are comparing ADMIXTURE estimates of African ancestry in the Caribbean Island individuals for Moreno-Estrada et. al. 2013 to African ancestry estimates in the South American individuals. (PDF) [file pgen.1005602.s006.pdf]

## Autosomal Ancestry

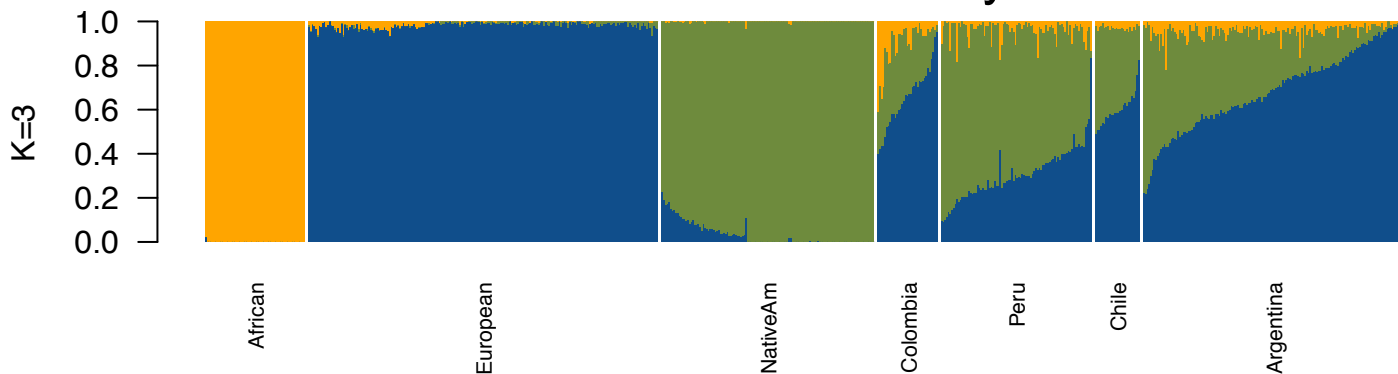

## X-chromosomal Ancestry

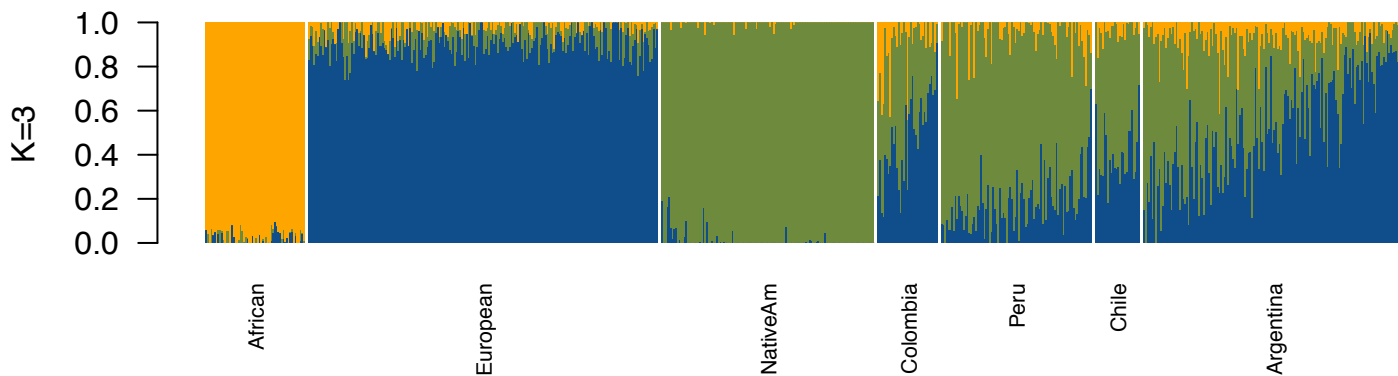

Supplement: S7 Fig — K = 3 ADMIXTURE proportions for admixed female individuals combined with a European, African, and Native American reference panel on autosomal chromosomes and on X-chromosomal markers. (PDF) [file pgen.1005602.s007.pdf]

**Colombia**

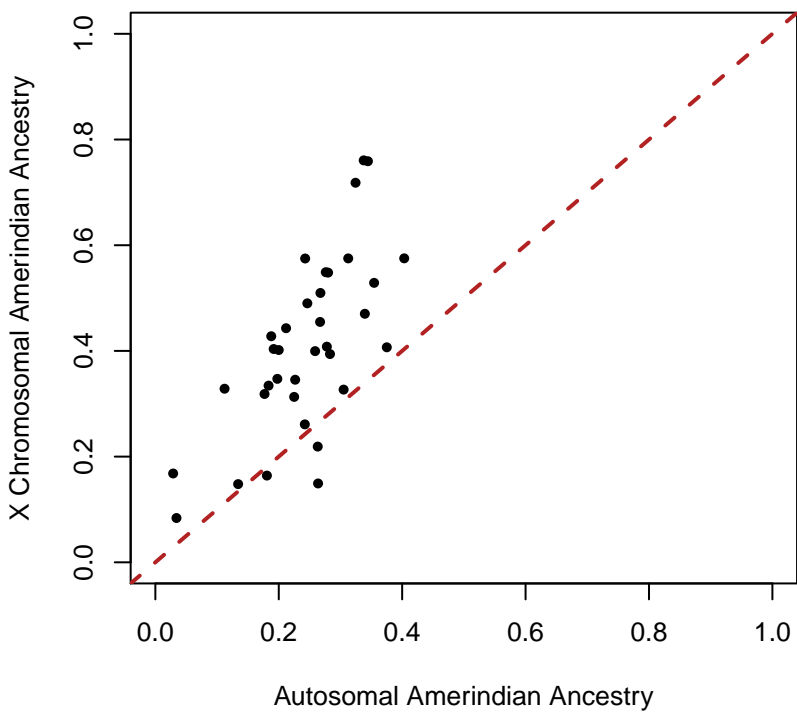

**Peru**

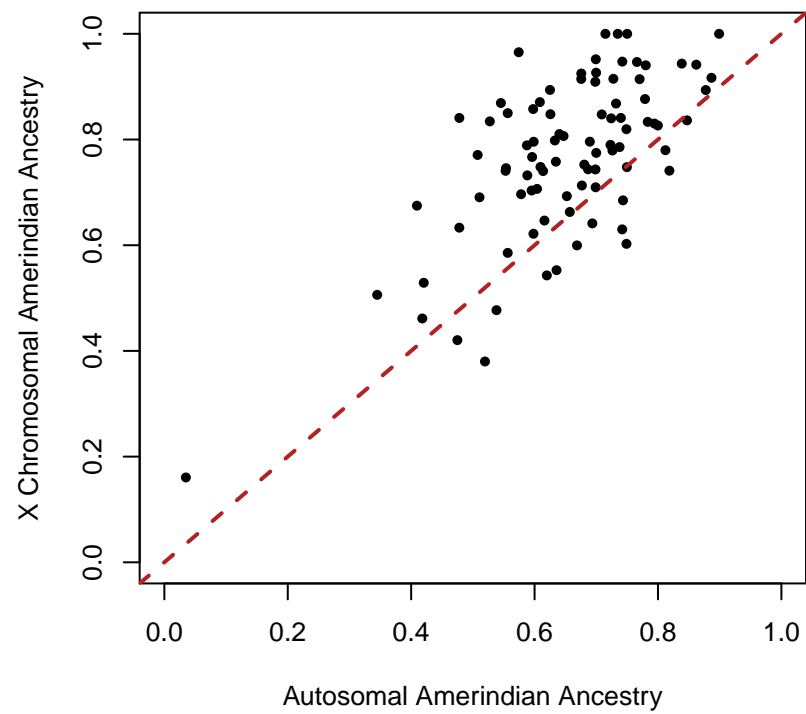

**Argentina**

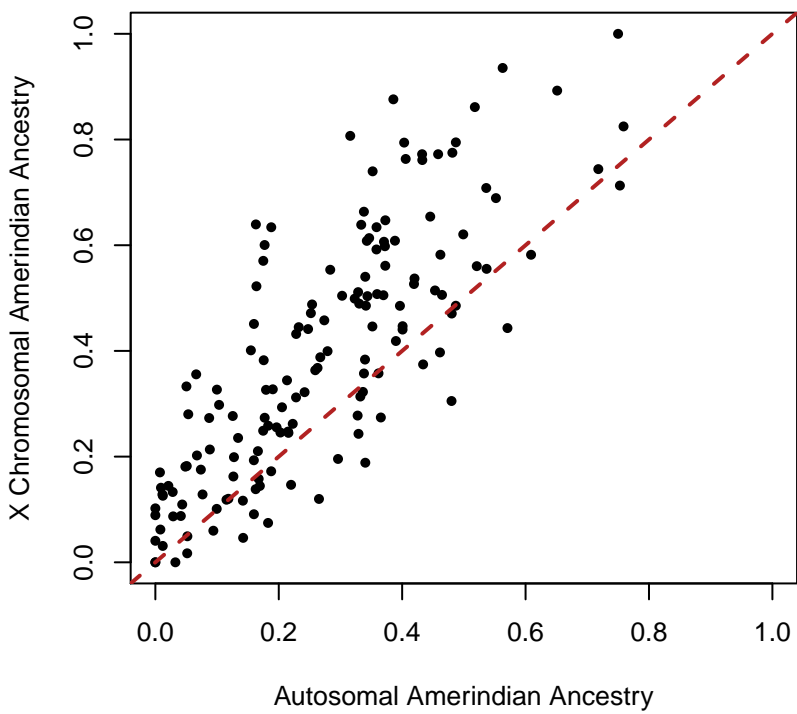

**Chile**

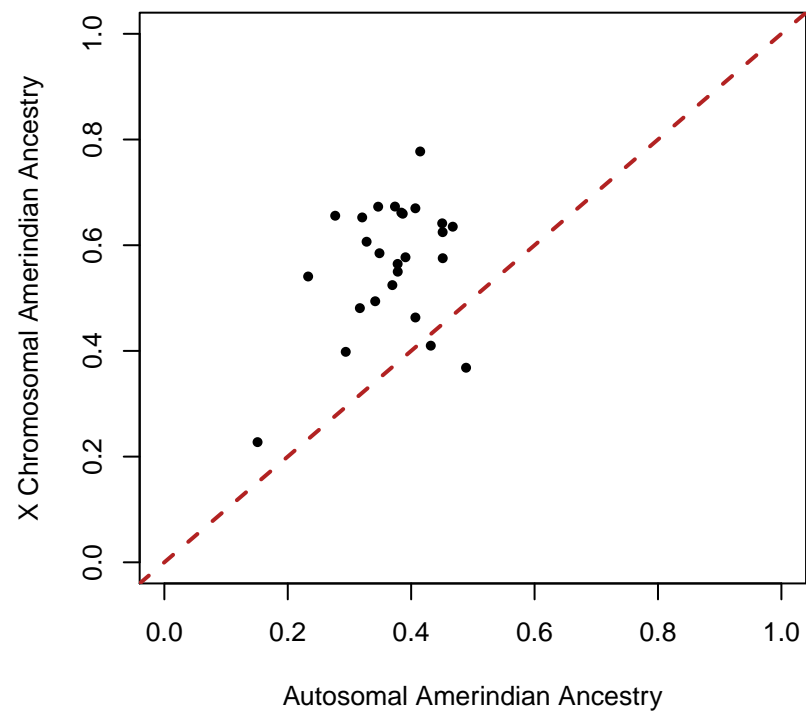

Supplement: S8 Fig — (PDF) [file pgen.1005602.s008.pdf]

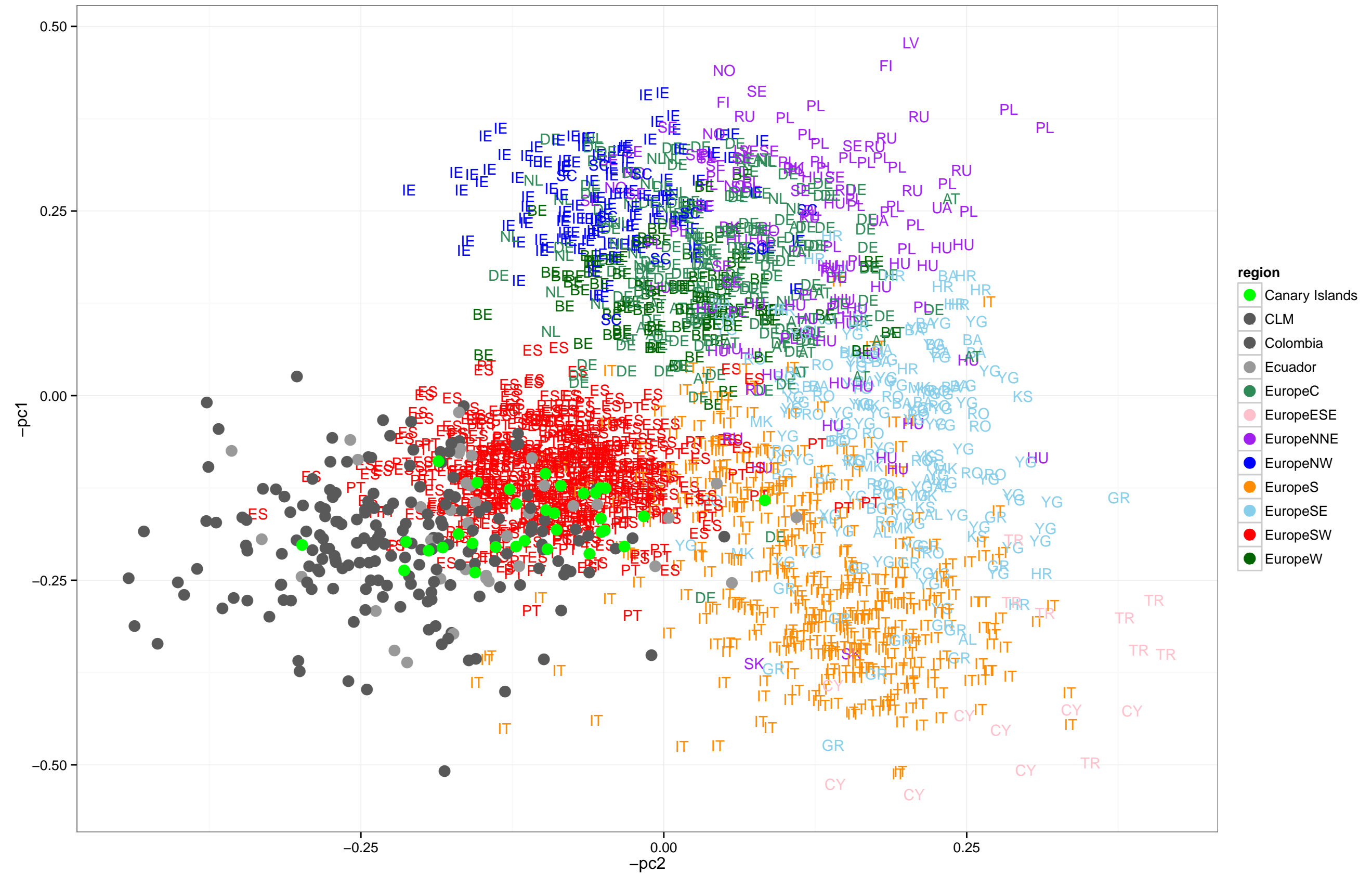

Supplement: S9 Fig — European ASPCA analysis of masked Colombians, Ecuadorians, Canary Islanders and the subcontinental POPRES reference. (PDF) [file pgen.1005602.s009.pdf]

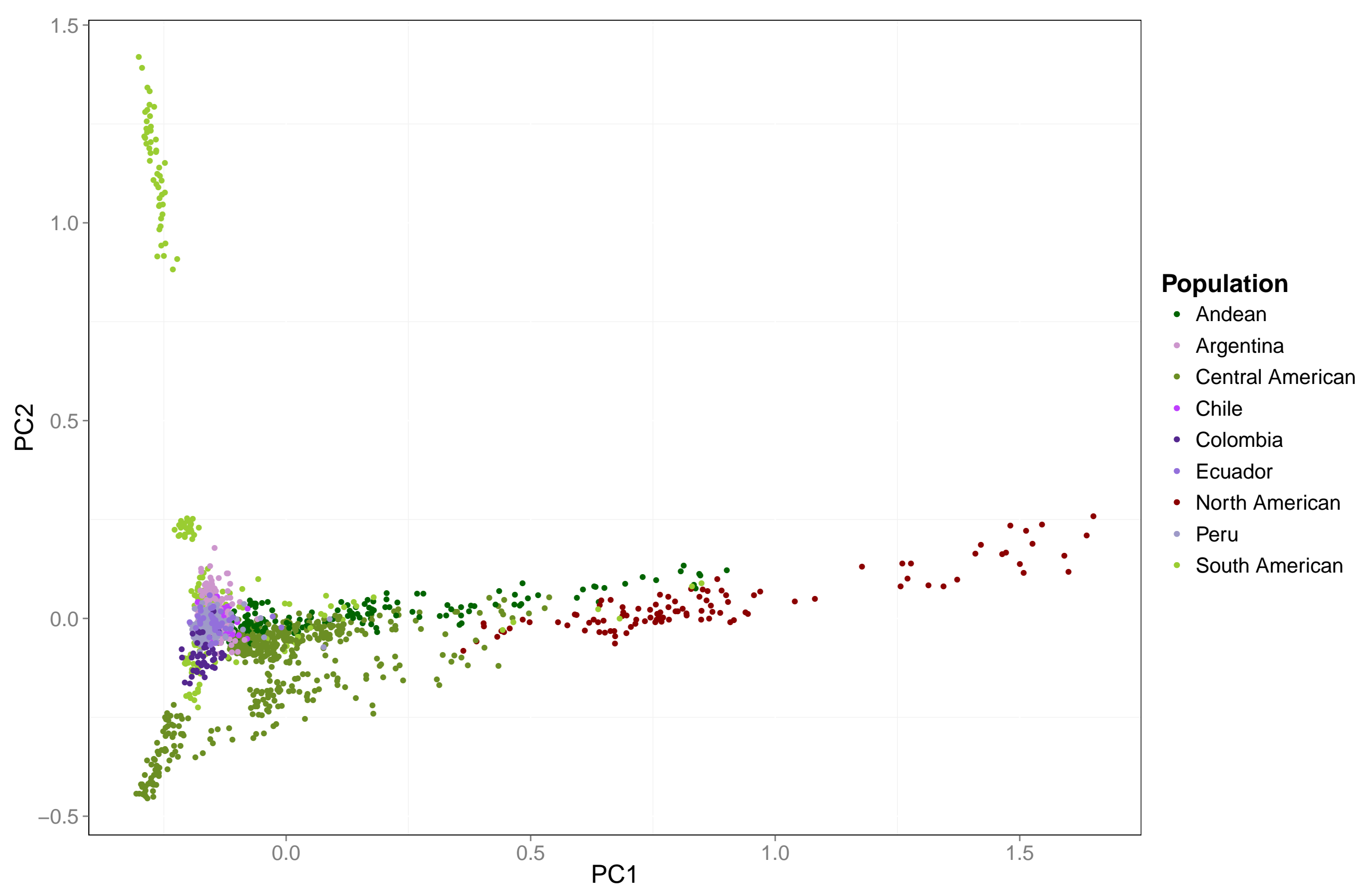

Supplement: S10 Fig — Admixed individuals are in purple while Native American individuals are in greens and reds. Outlier populations (see outlying clusters on PC2) and Eskimo-Aleut and Na-Dene populations were removed from the analysis. (PDF) [file pgen.1005602.s010.pdf]

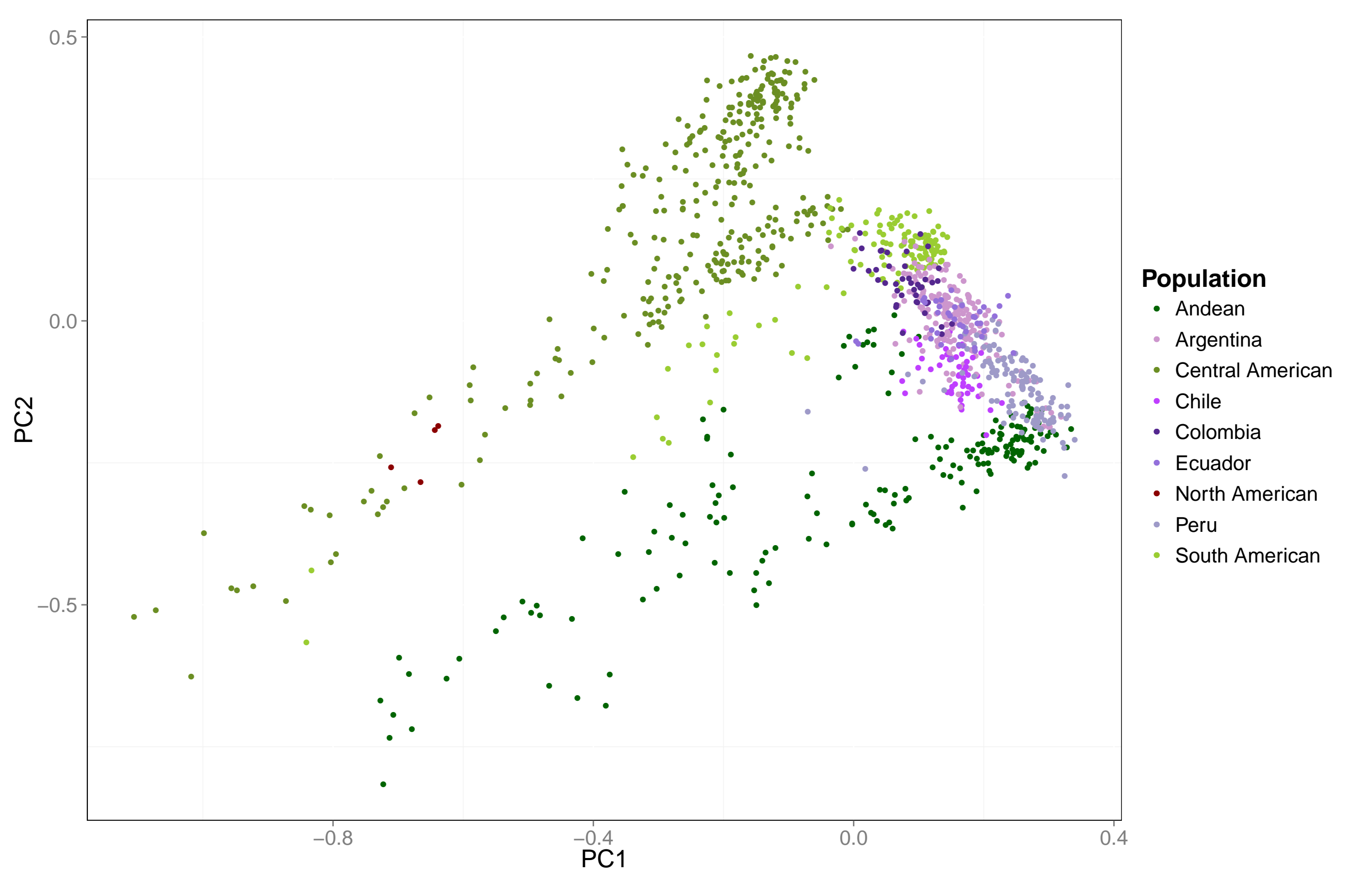

Supplement: S11 Fig — Admixed individuals are in purple and Native American individuals are in greens. The spread towards the bottom left corner is due to European admixture (see S8 and S9 Fig). (PDF) [file pgen.1005602.s011.pdf]

## European Ancestry vs ASPCA1 Native Americans

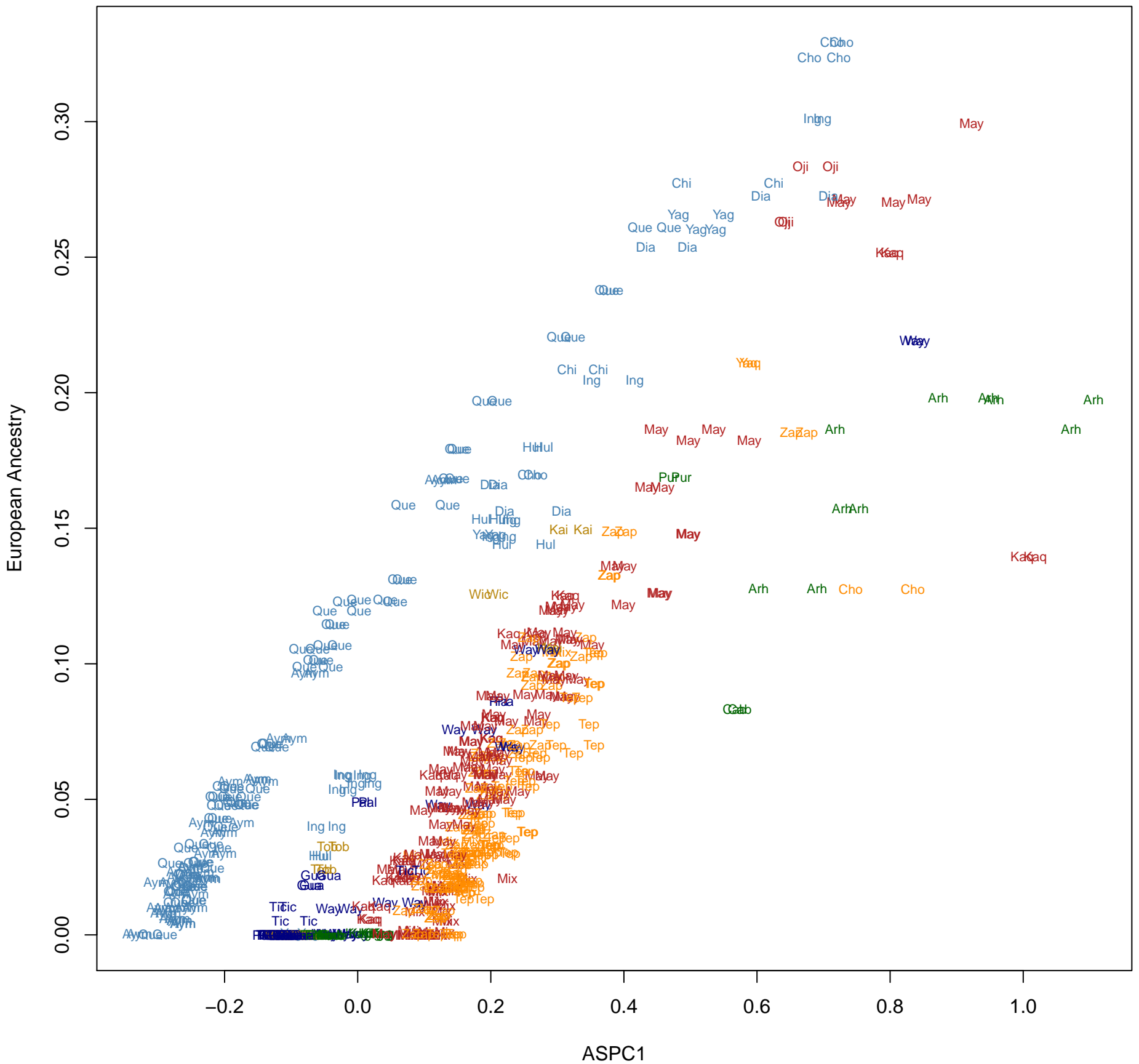

Supplement: S12 Fig — Proportion of European ancestry (estimated through ADMIXTURE at K = 4) in unmasked Native American Samples vs. position on ASPC1 in S7 Fig. (PDF) [file pgen.1005602.s012.pdf]

# European Ancestry vs ASPCA2 Native Americans

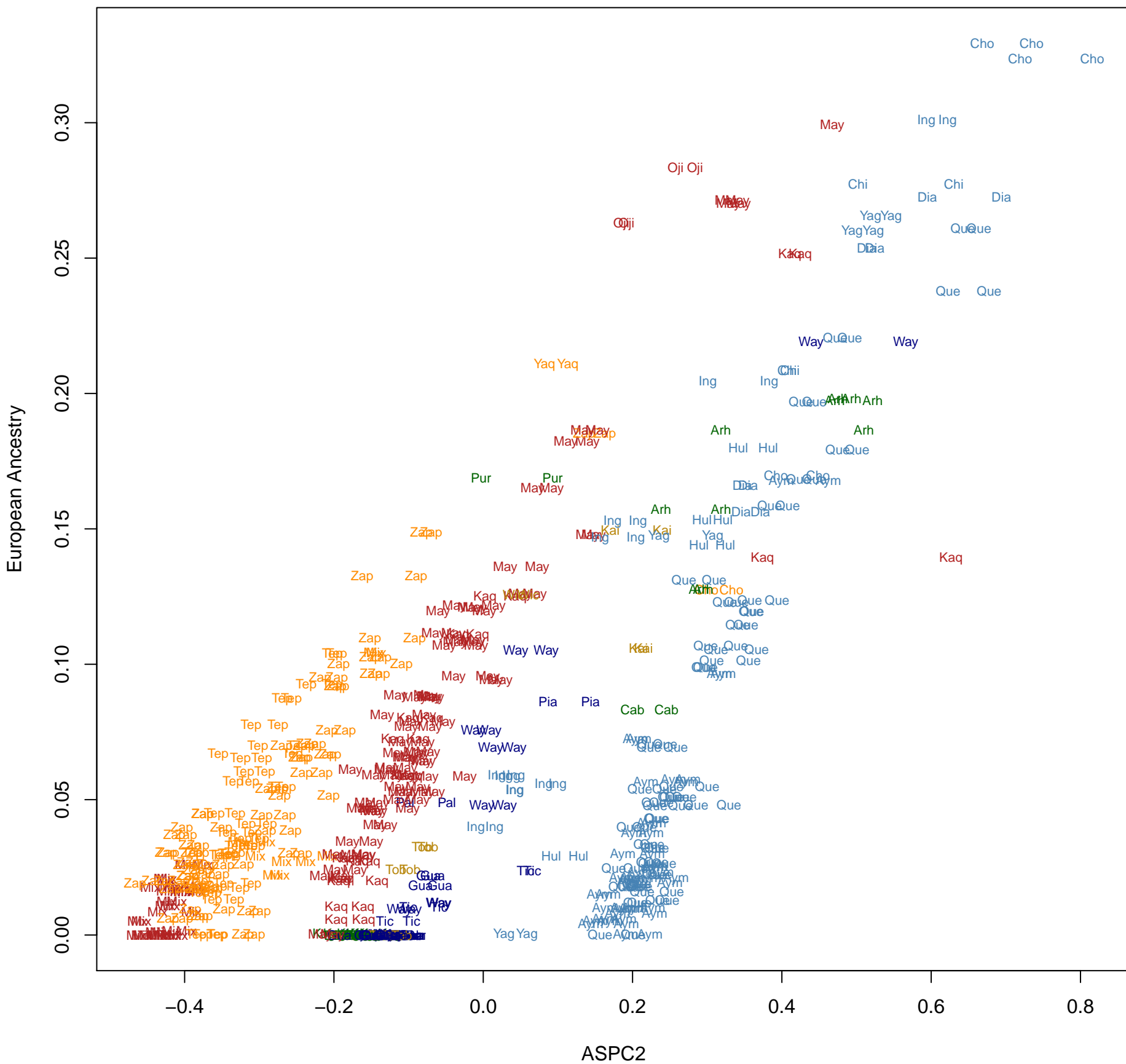

Supplement: S13 Fig — Proportion of European ancestry (estimated through ADMIXTURE at K = 4) in unmasked Native American Samples vs. position on ASPC2 in S7 Fig. (PDF) [file pgen.1005602.s013.pdf]

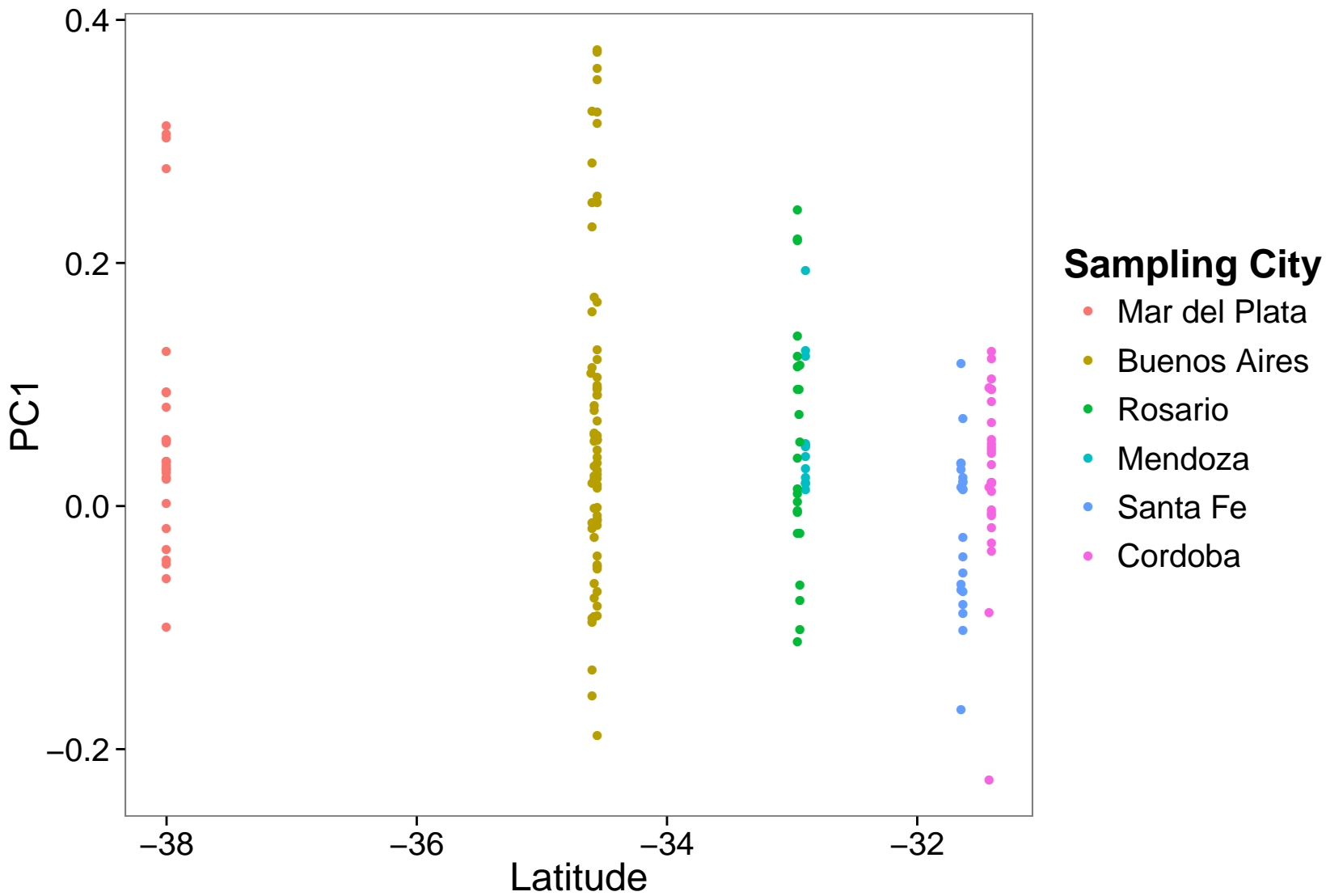

Supplement: S14 Fig — Colors correspond to the sampling city. (PDF) [file pgen.1005602.s014.pdf]

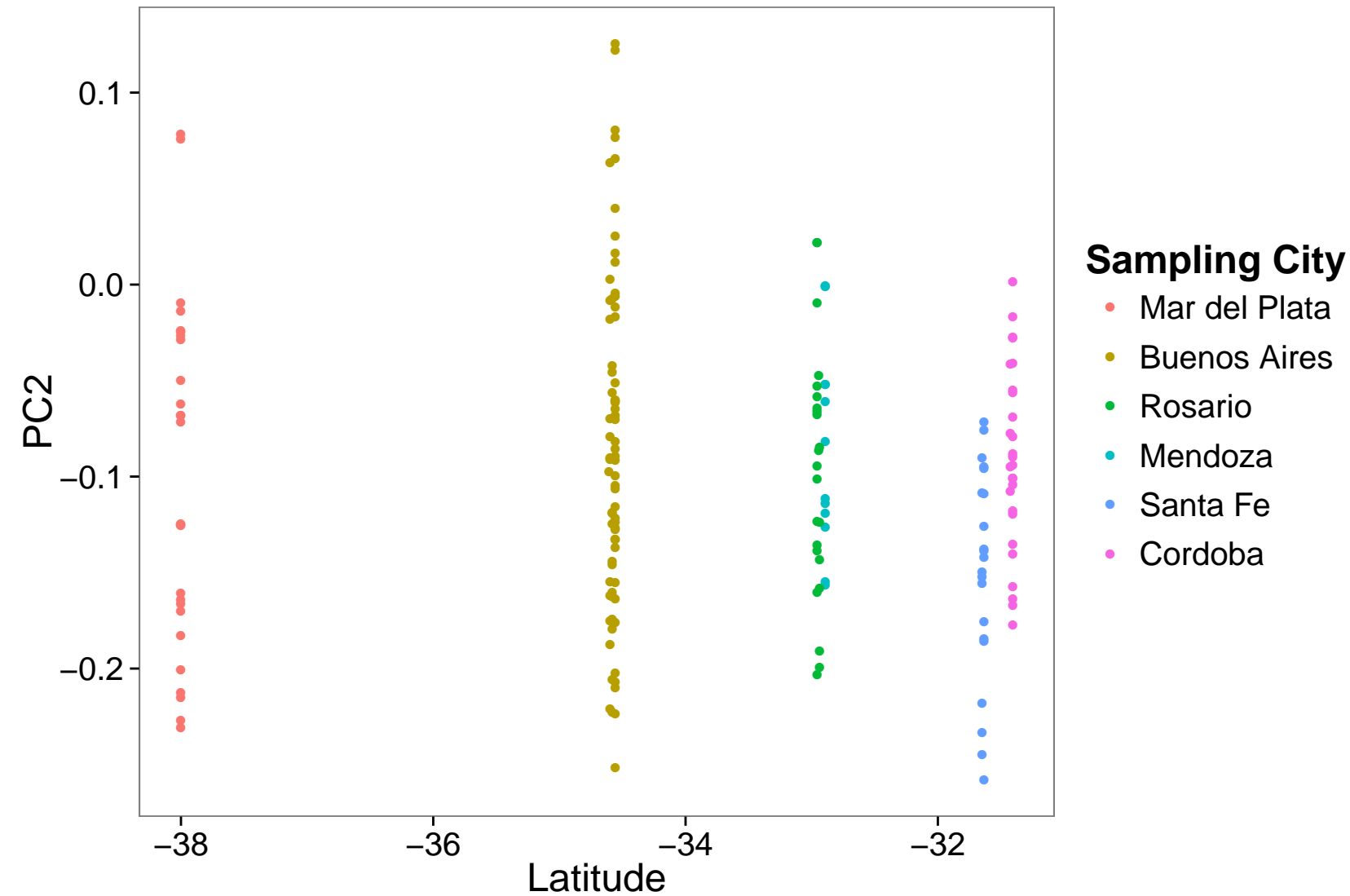

Supplement: S15 Fig — Colors correspond to the sampling city. (PDF) [file pgen.1005602.s015.pdf]

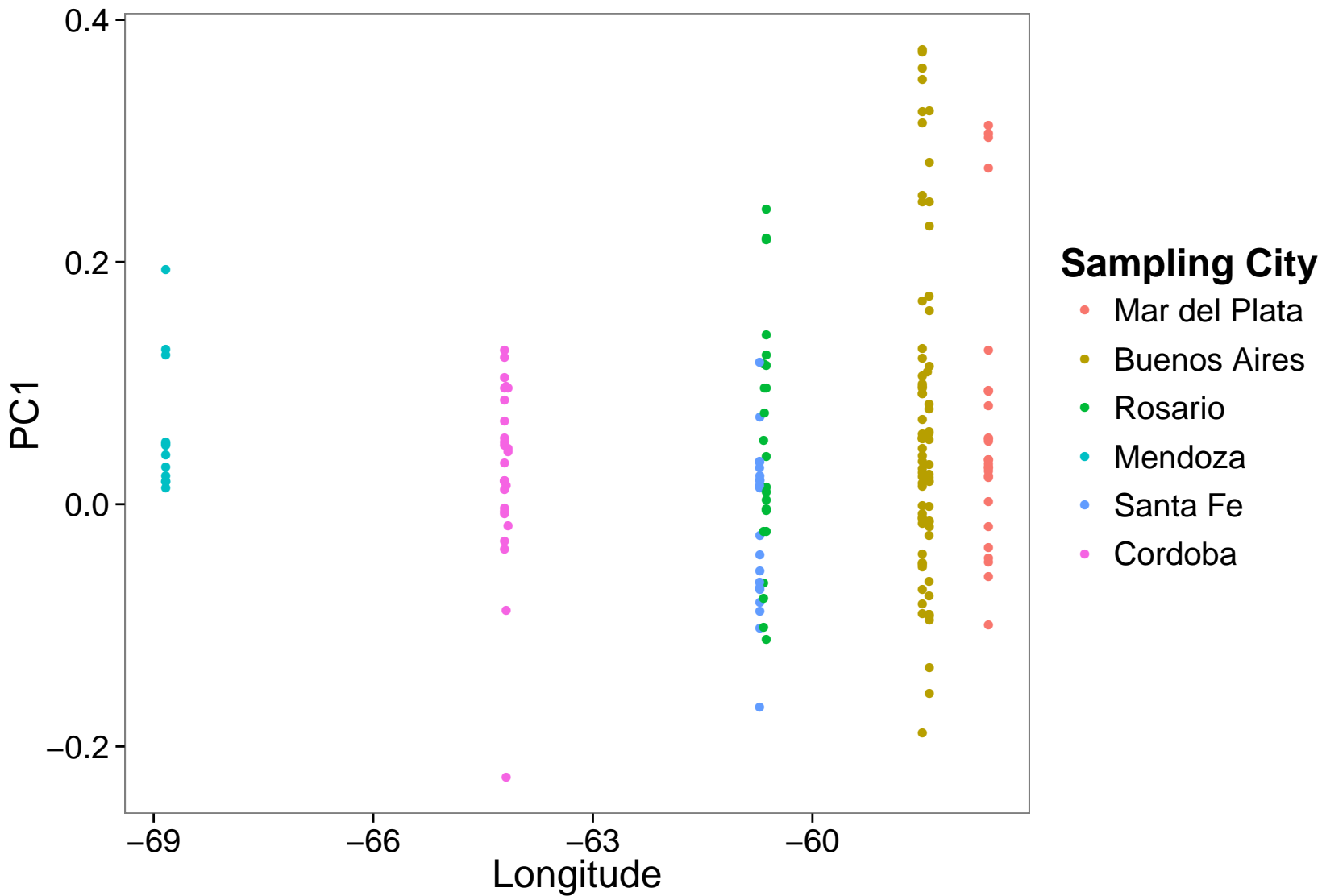

Supplement: S16 Fig — Colors correspond to the sampling city. (PDF) [file pgen.1005602.s016.pdf]

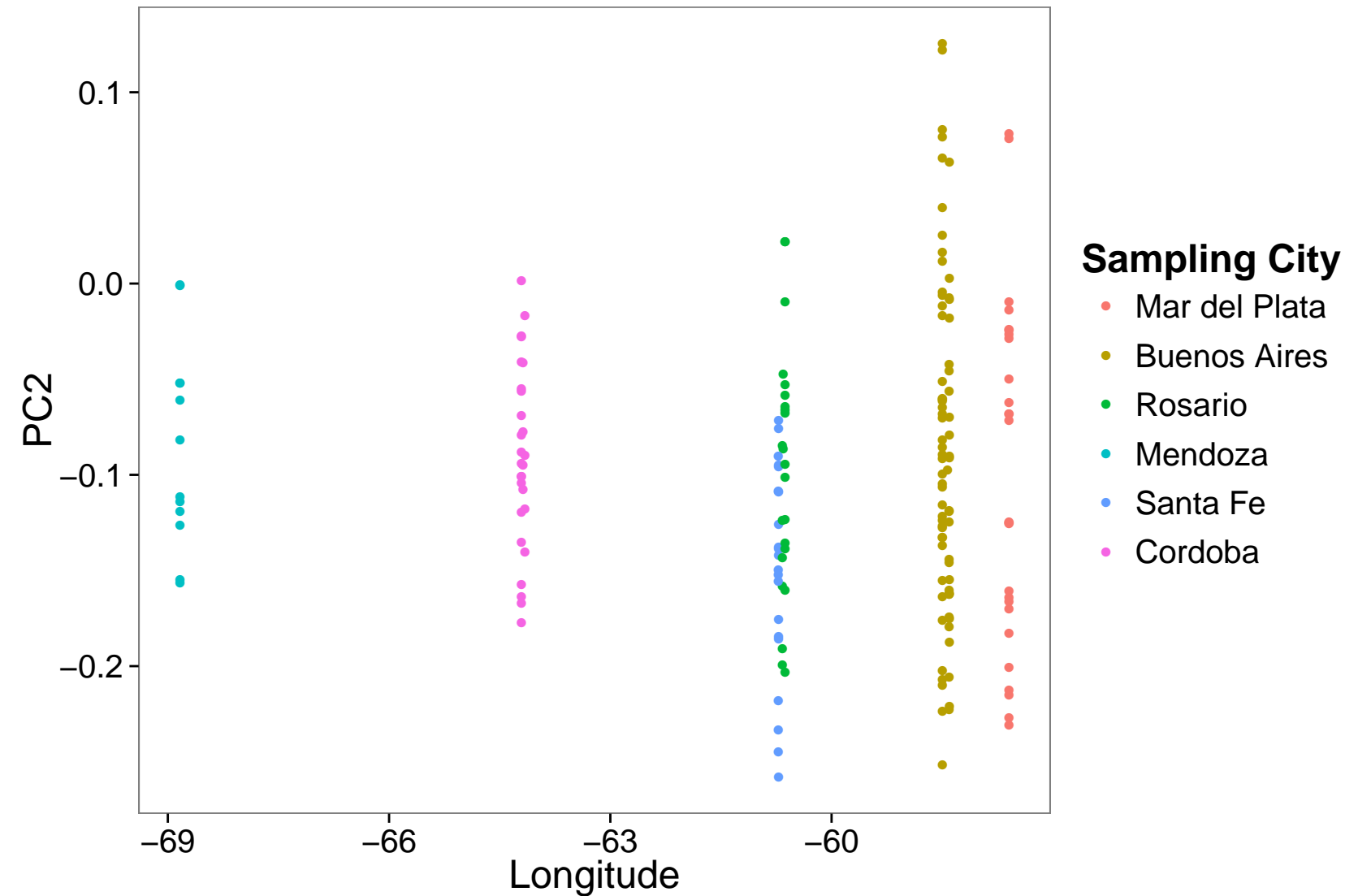

Supplement: S17 Fig — Colors correspond to the sampling city. (PDF) [file pgen.1005602.s017.pdf]

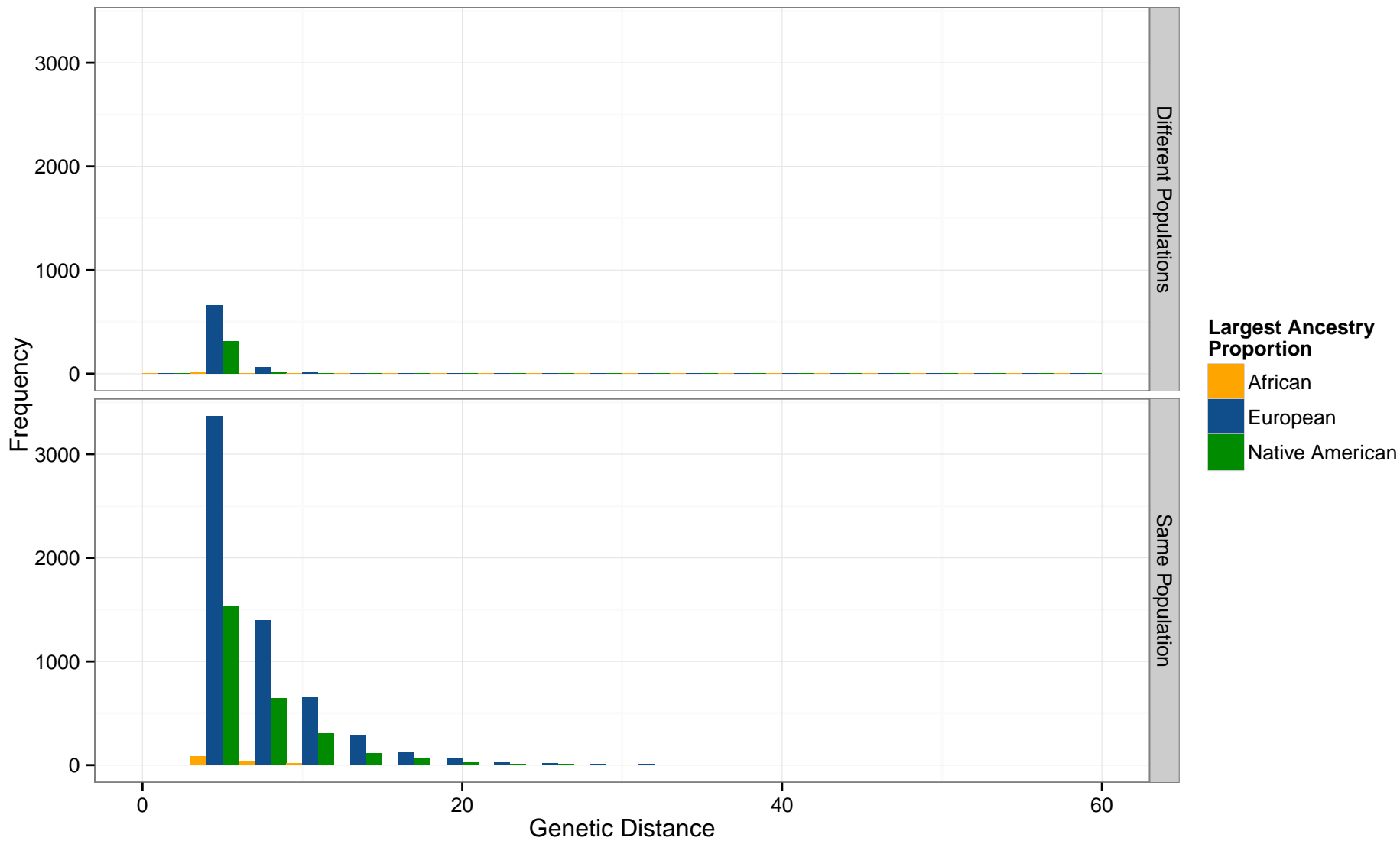

Supplement: S18 Fig — Histogram of IBD tracts shared within and between populations by the most common ancestry within each IBD tract. (PDF) [file pgen.1005602.s018.pdf]

Proportion of European Ancestry in IBD Tracts Shared Between Populations

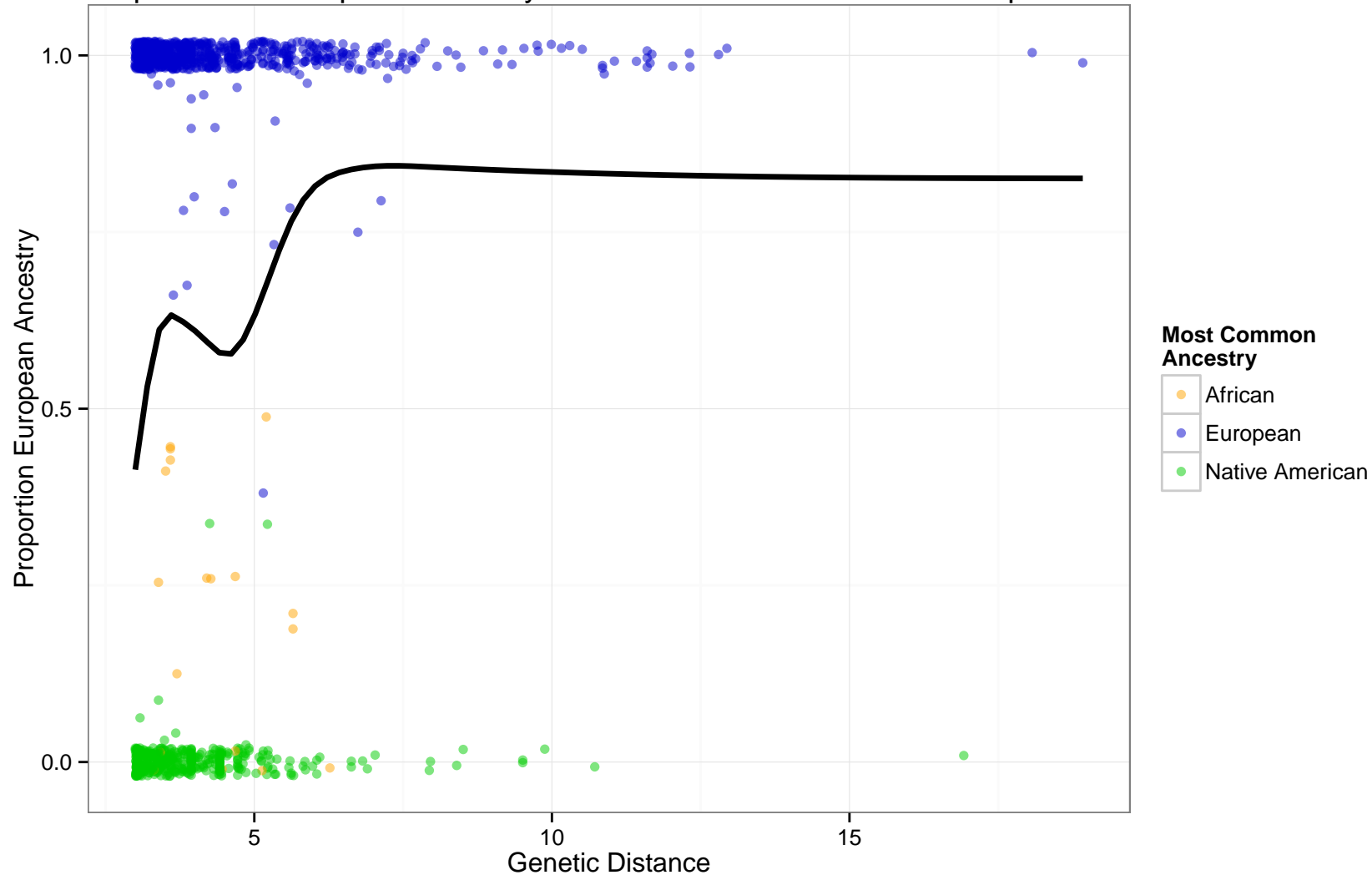

Supplement: S19 Fig — Comparison of the proportion of European ancestry in an IBD tract compared with IBD tract length for tracts shared between populations. (PDF) [file pgen.1005602.s019.pdf]

Proportion of European Ancestry in IBD Tracts Shared Within Populations

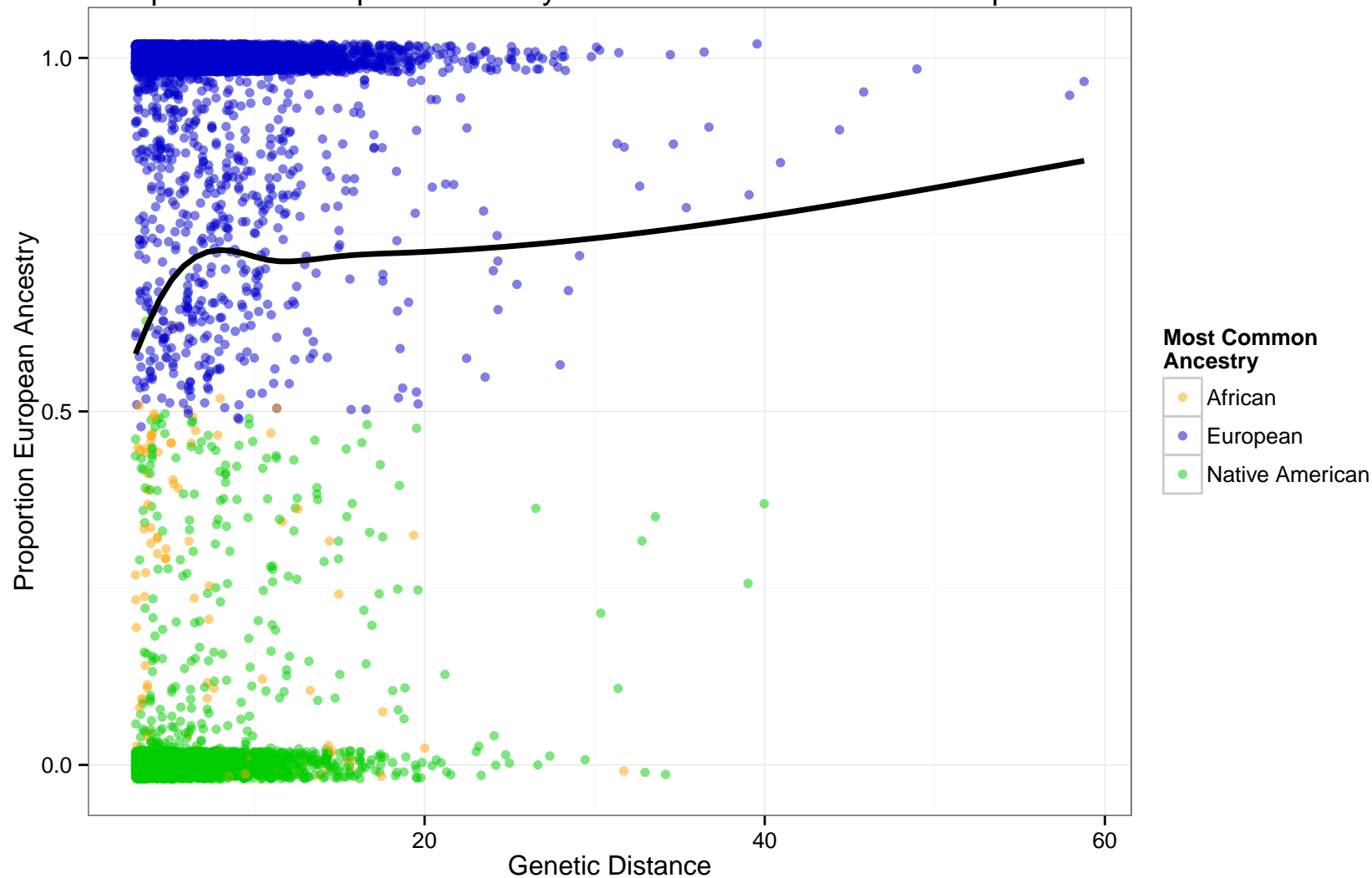

Supplement: S20 Fig — Comparison of the proportion of European ancestry in an IBD tract compared with IBD tract length for tracts shared within populations. (PDF) [file pgen.1005602.s020.pdf]

IBD matches with European Populations

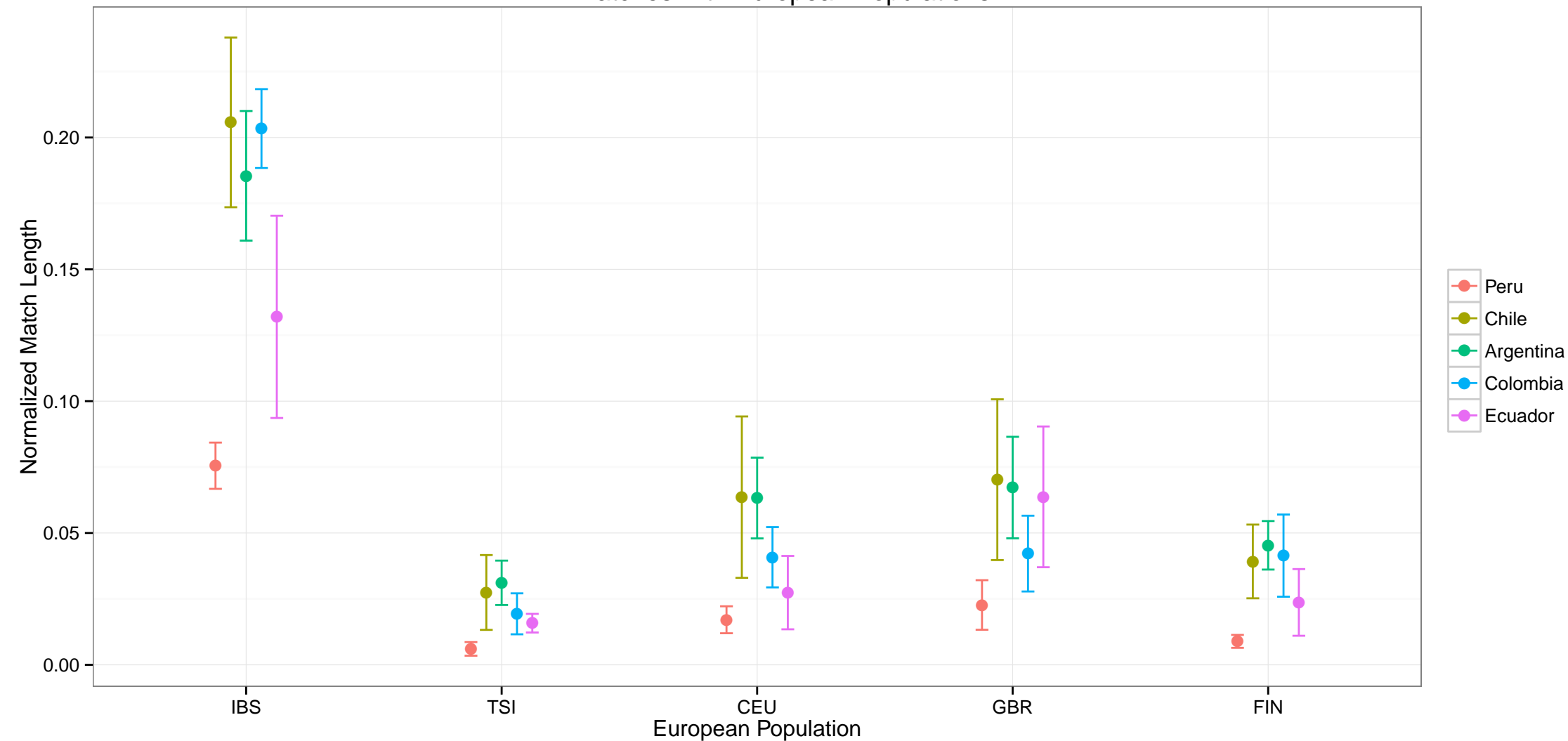

Supplement: S22 Fig — Normalized length of IBD matches between admixed populations and European populations from 1000 Genomes. For each population, the calculated value and jackknife standard error bars are shown. (PDF) [file pgen.1005602.s022.pdf]

IBD matches with Native Populations

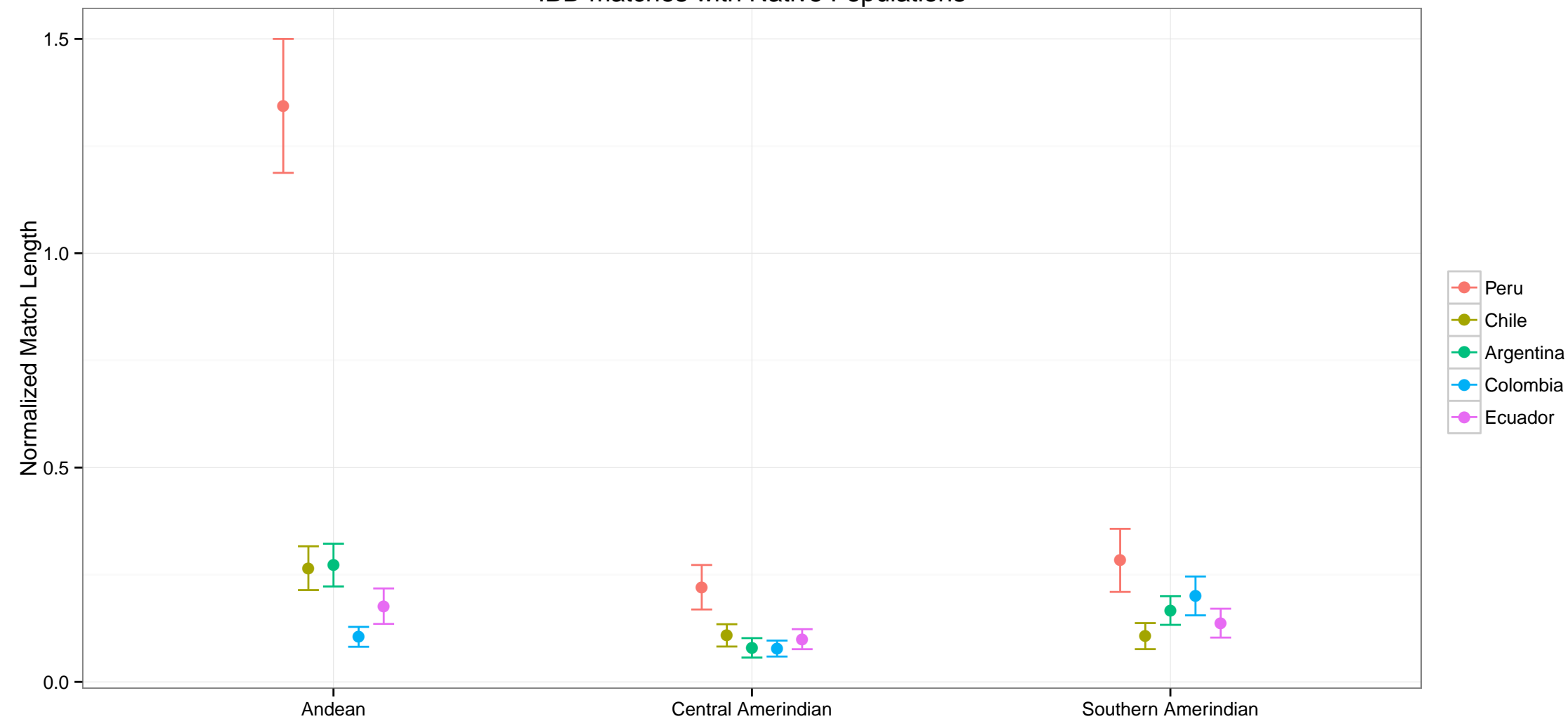

Supplement: S23 Fig — Normalized length of IBD matches between admixed populations and Native American populations. For each population, the calculated value and jackknife standard error bars are shown. (PDF) [file pgen.1005602.s023.pdf]

Argentina

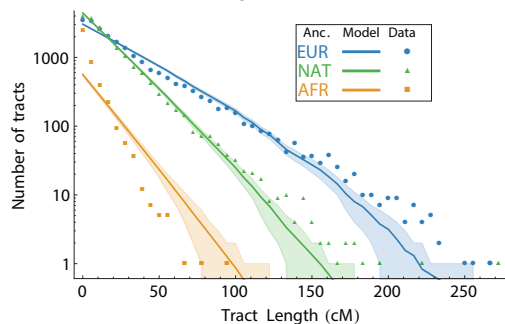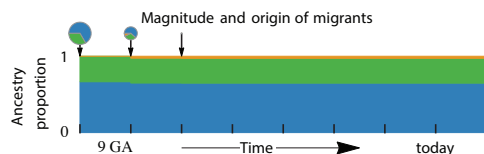

Chile

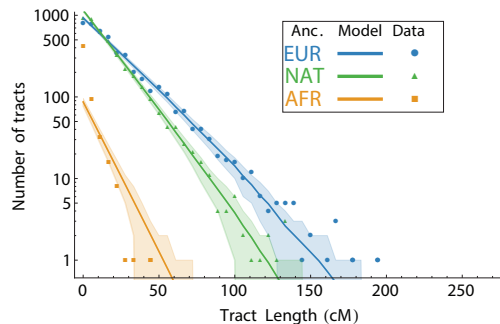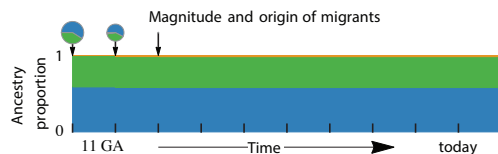

Colombia

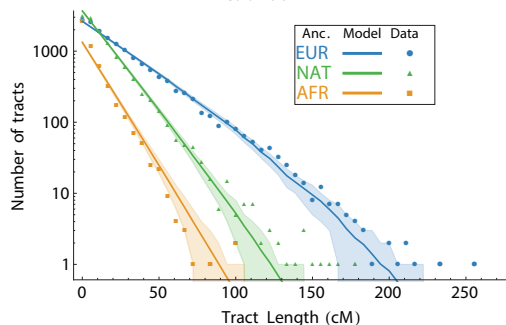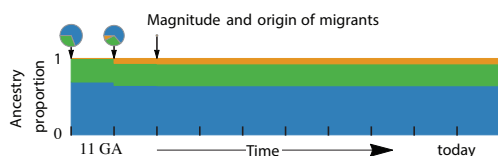

Ecuador

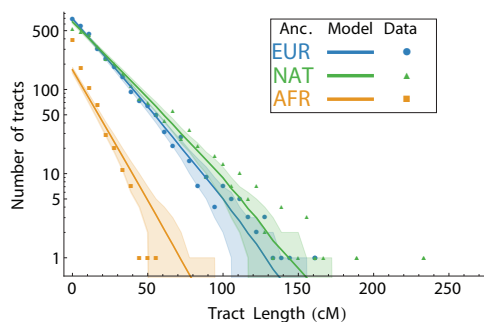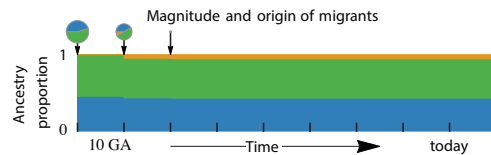

Peru

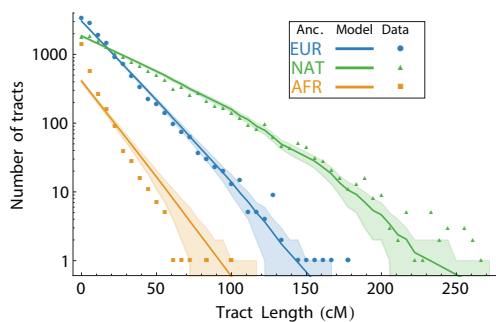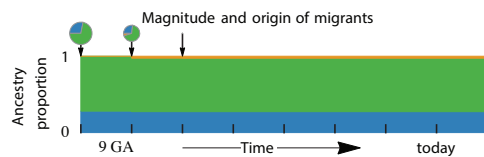

Supplement: S25 Fig — Tracts Analysis results for the base model, which has a European/Native American admixture event followed by a pulse of African migration. Results are shown for each of the countries. The first half of each panel plots the distribution of ancestry tract lengths found within the individuals. The line plots the expected distribution given the fitted Tracts models, with the shading indicating the 68% confidence interval. The second plot on each panel shows the change in ancestry proportions over time within the admixed populations. Tracts of specific ancestries are colored as follows: blue = European, green = Native American, orange = African. (PDF) [file pgen.1005602.s025.pdf]

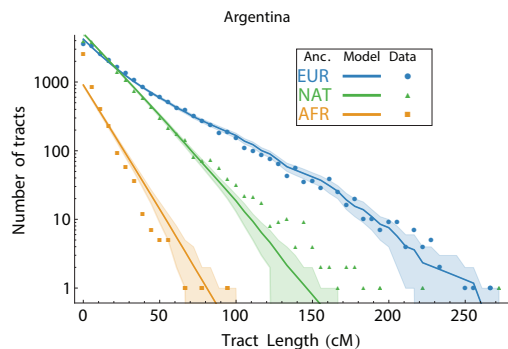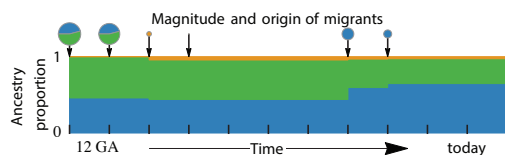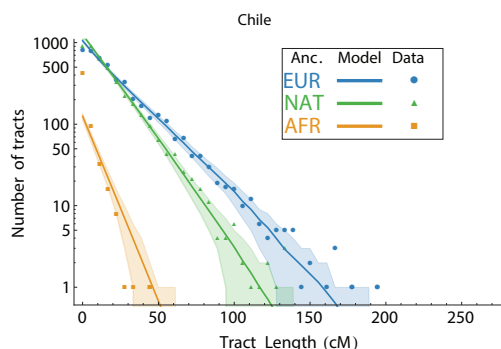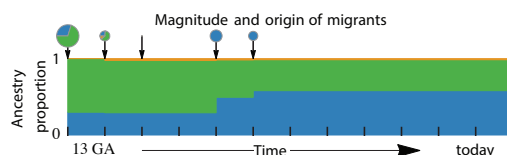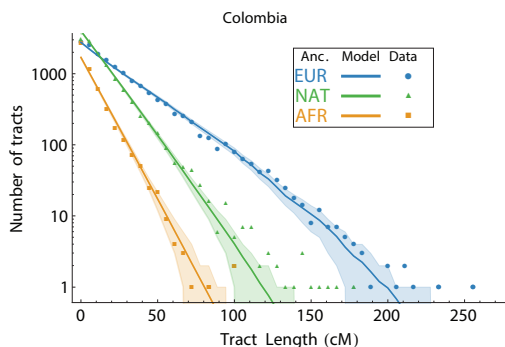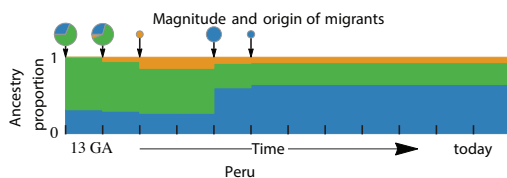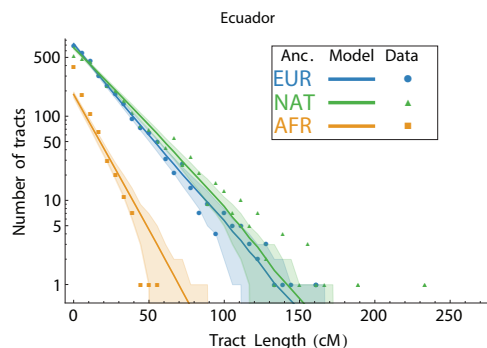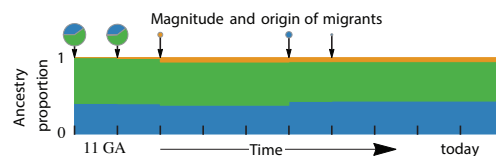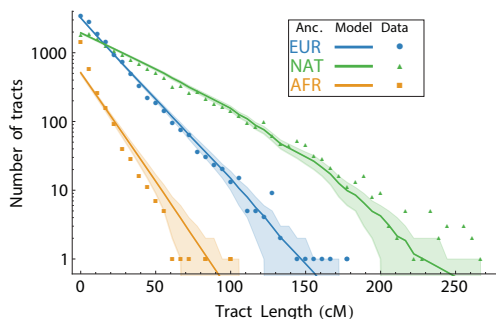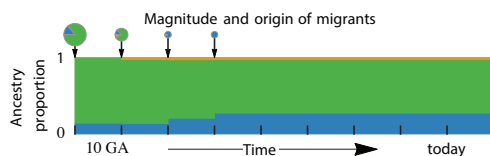

Supplement: S26 Fig — Tracts Analysis results for the Tracts model with an additional pulse of European ancestry. Results are shown for each of the countries. The first half of each panel plots the distribution of ancestry tract lengths found within the individuals. The line plots the expected distribution given the fitted Tracts models, with the shading indicating the 68% confidence interval. The second plot on each panel shows the change in ancestry proportions over time within the admixed populations. Tracts of specific ancestries are colored as follows: blue = European, green = Native American, orange = African. (PDF) [file pgen.1005602.s026.pdf]

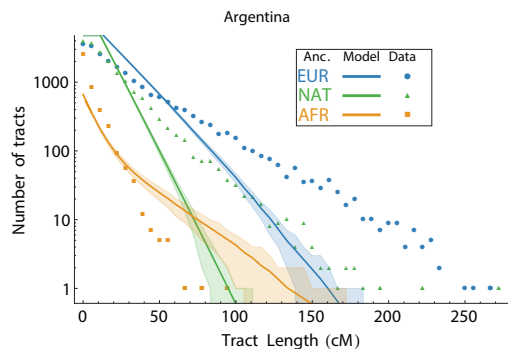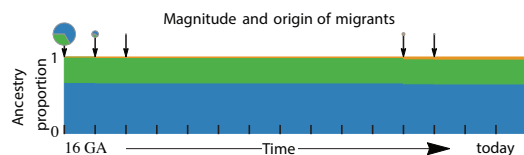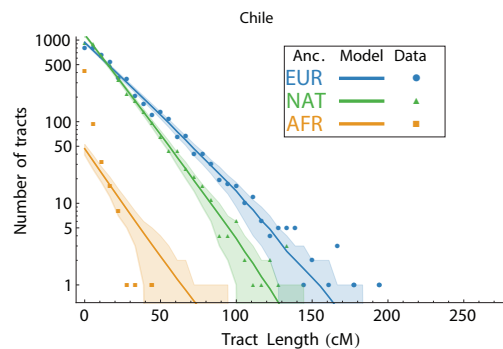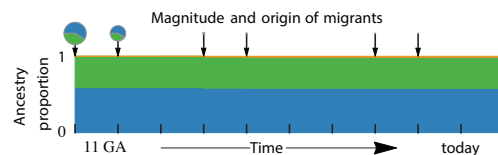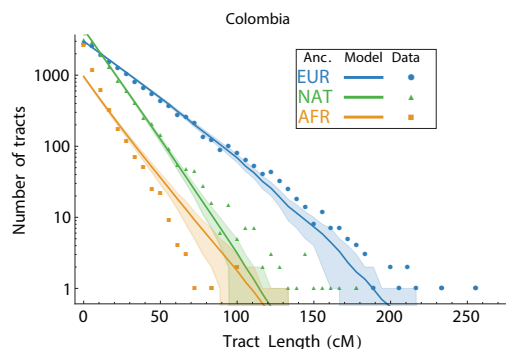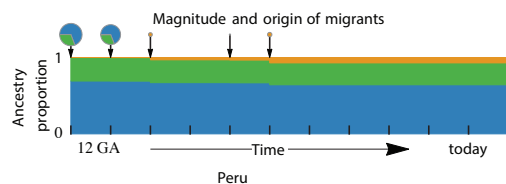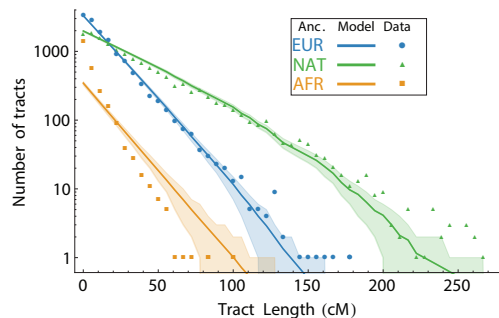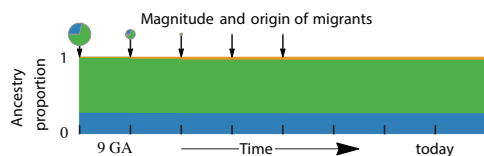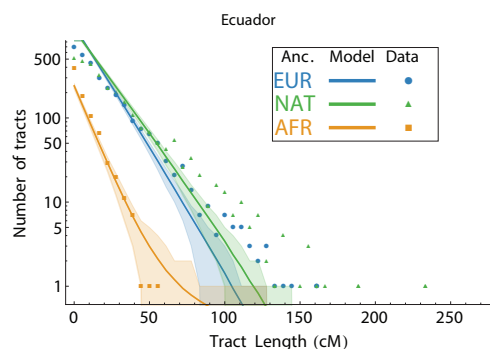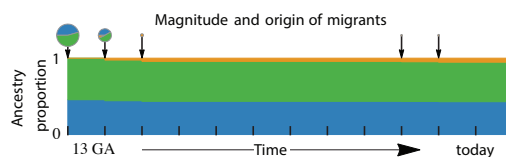

Supplement: S27 Fig — Tracts Analysis results for the Tracts model with an additional pulse of African ancestry. Results are shown for each of the countries. The first half of each panel plots the distribution of ancestry tract lengths found within the individuals. The line plots the expected distribution given the fitted Tracts models, with the shading indicating the 68% confidence interval. The second plot on each panel shows the change in ancestry proportions over time within the admixed populations. Tracts of specific ancestries are colored as follows: blue = European, green = Native American, orange = African. (PDF) [file pgen.1005602.s027.pdf]

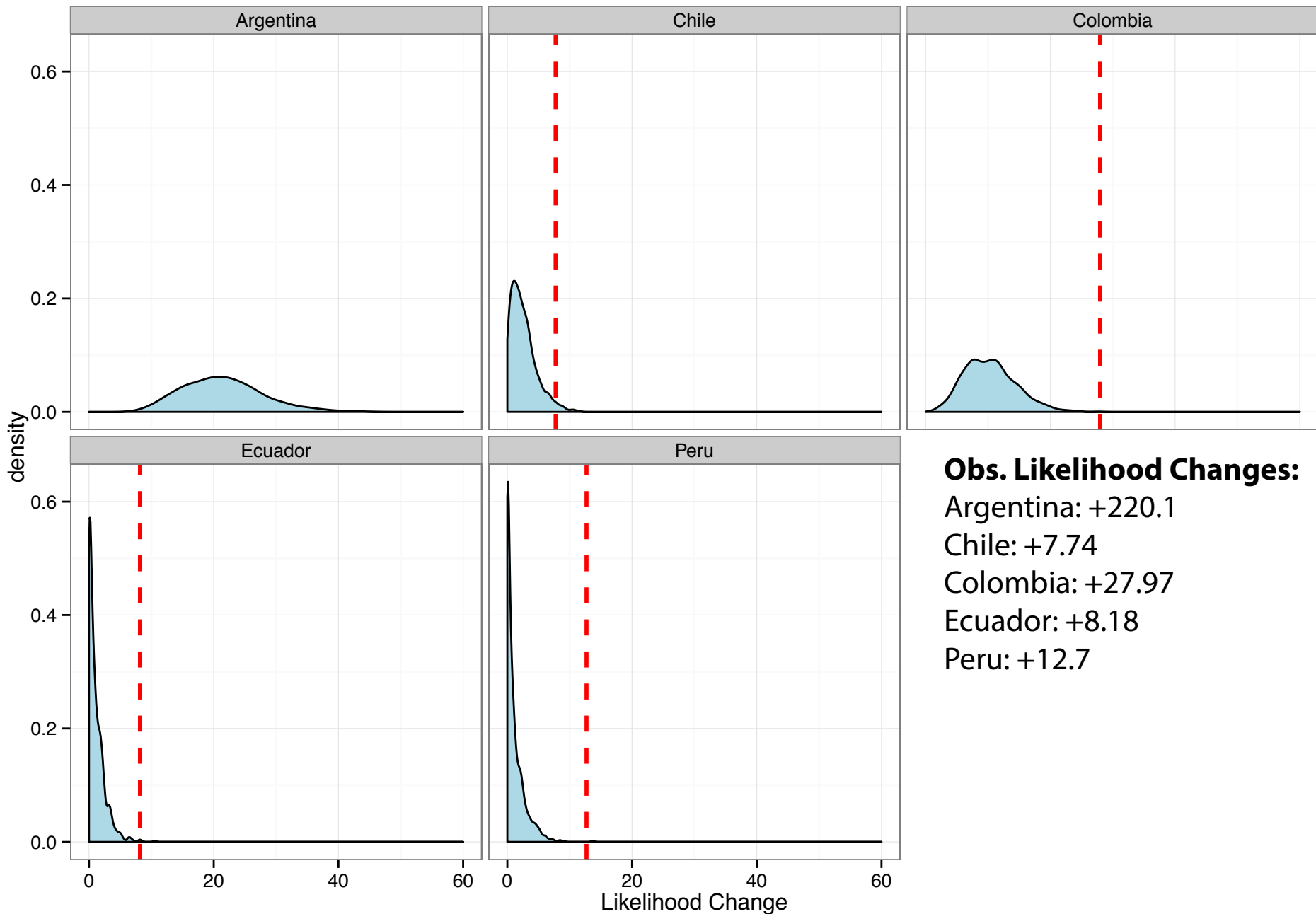

Supplement: S28 Fig — Here we have plotted the simulated likelihood differences between a single pulse and double pulse model of admixture for each population. The dashed red line indicates the observed likelihood change for each population. Note that for Argentina, the observed likelihood change (+220.1) was so great that it is not plotted within the axis limits. We simulated 1000 tract length distributions for each population based upon the best fitting single pulse model. We then assessed the increase in likelihood in a simulated environment that occurred with the addition of an extra pulse of European migration. If our observed likelihood change was greater than 95% of the simulated likelihood changes, we preferred the model with the extra European migration pulse. (PDF) [file pgen.1005602.s028.pdf]

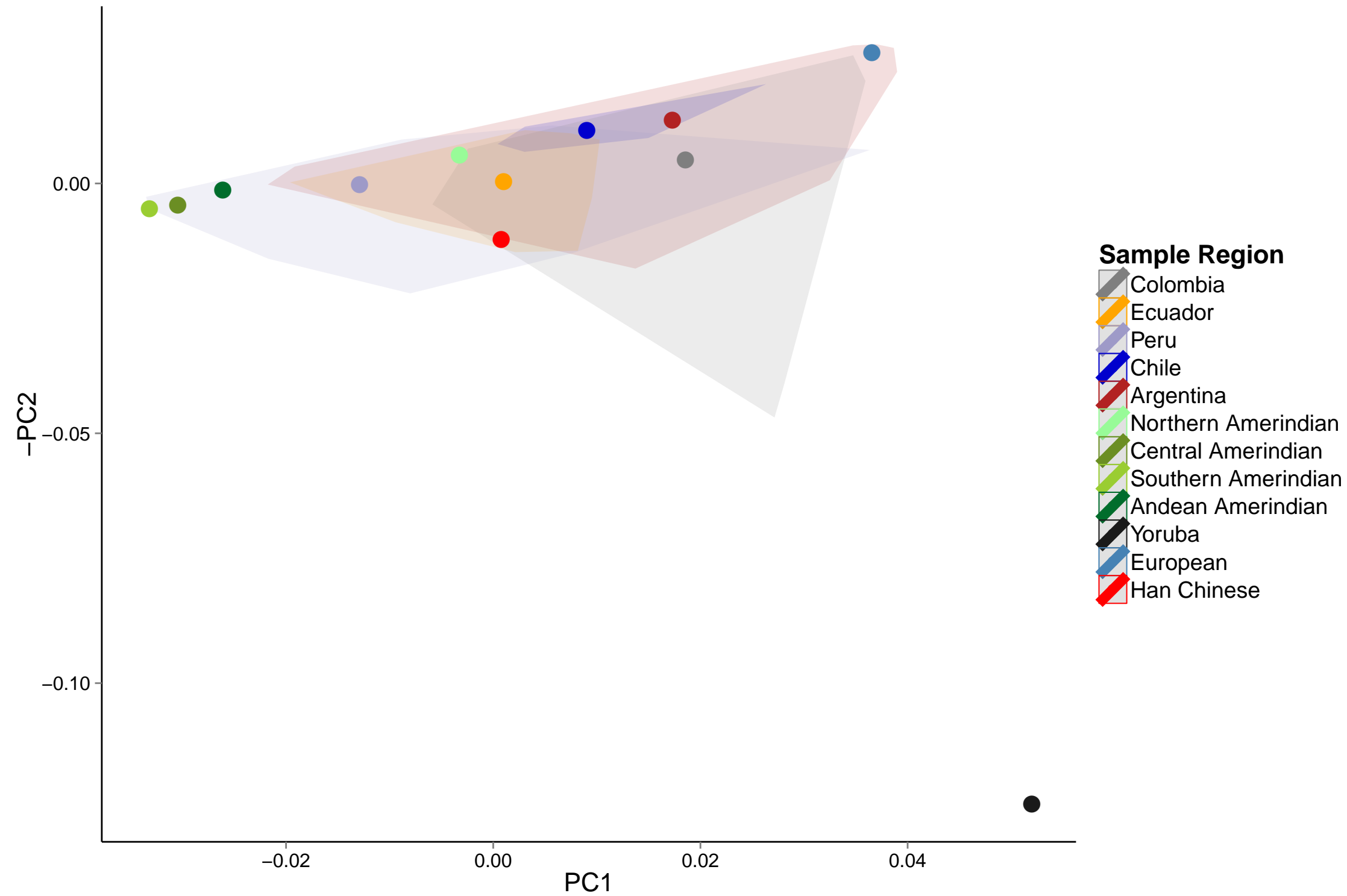

Supplement: S29 Fig — Population centroids and shaded regions are plotted for all of the populations included in the analysis. (PDF) [file pgen.1005602.s029.pdf]

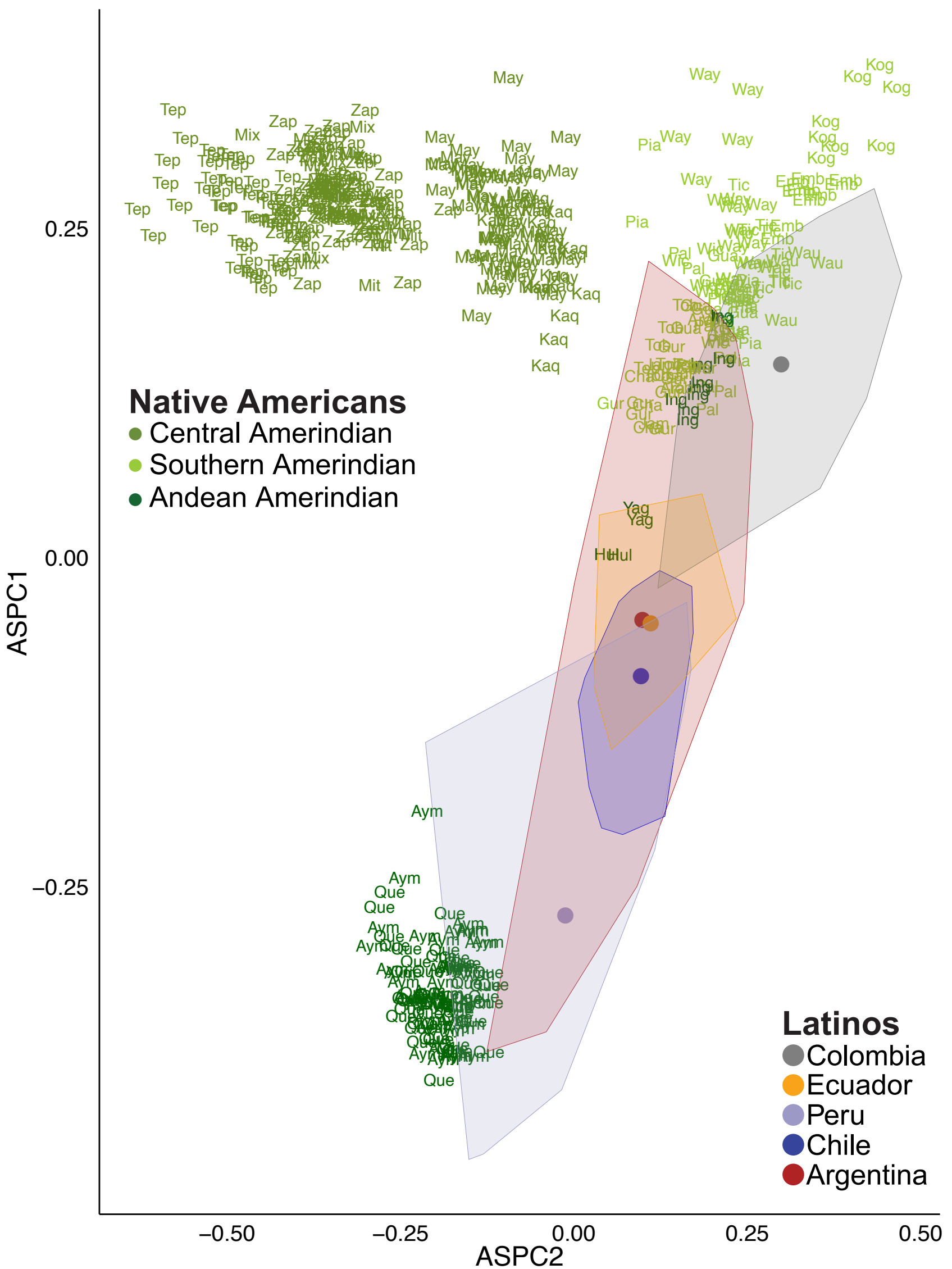

Supplement: S30 Fig — Population centroids and shaded regions are plotted for the admixed populations in the Native American ASPCA analysis. (PDF) [file pgen.1005602.s030.pdf]

Native American

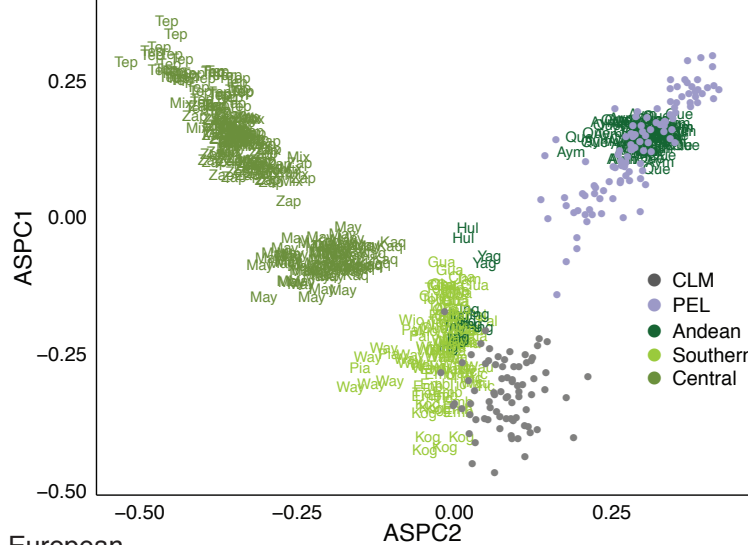

Pop Phased

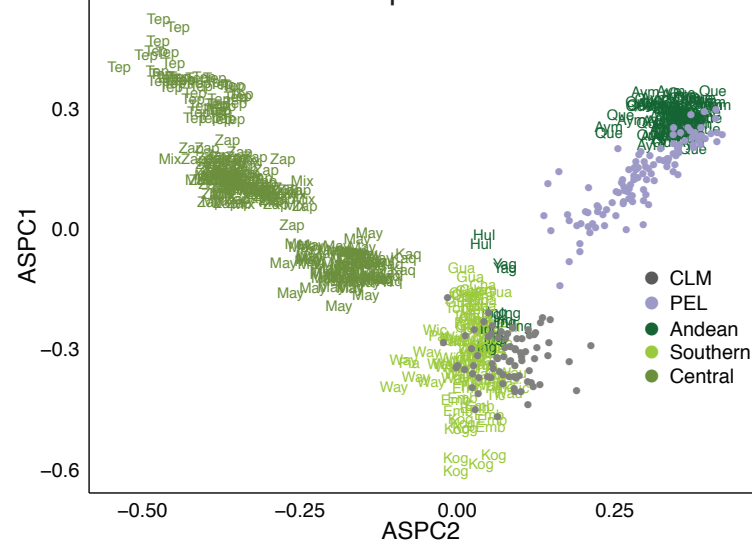

European

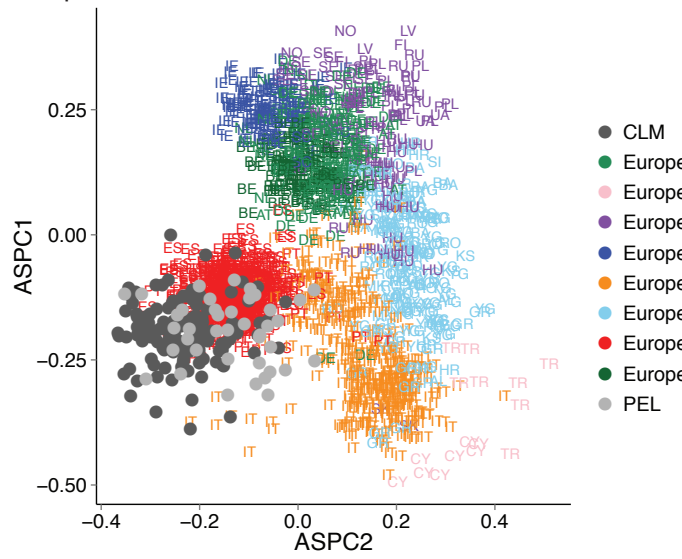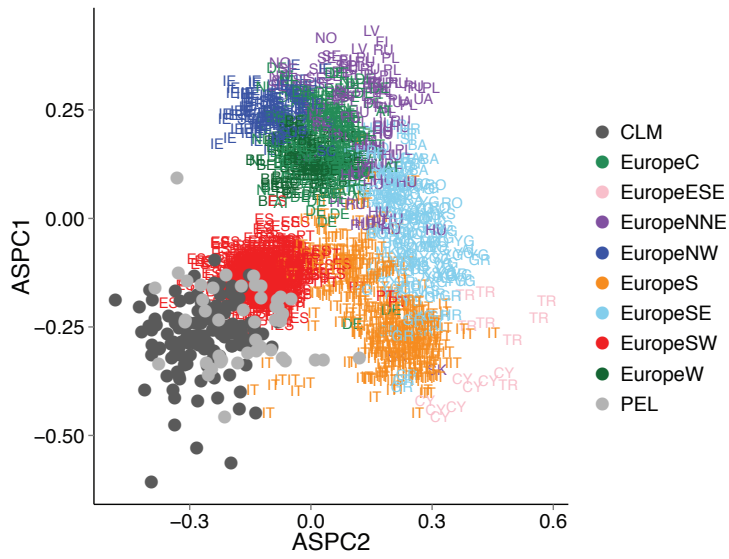

Supplement: S31 Fig — (PDF) [file pgen.1005602.s031.pdf]

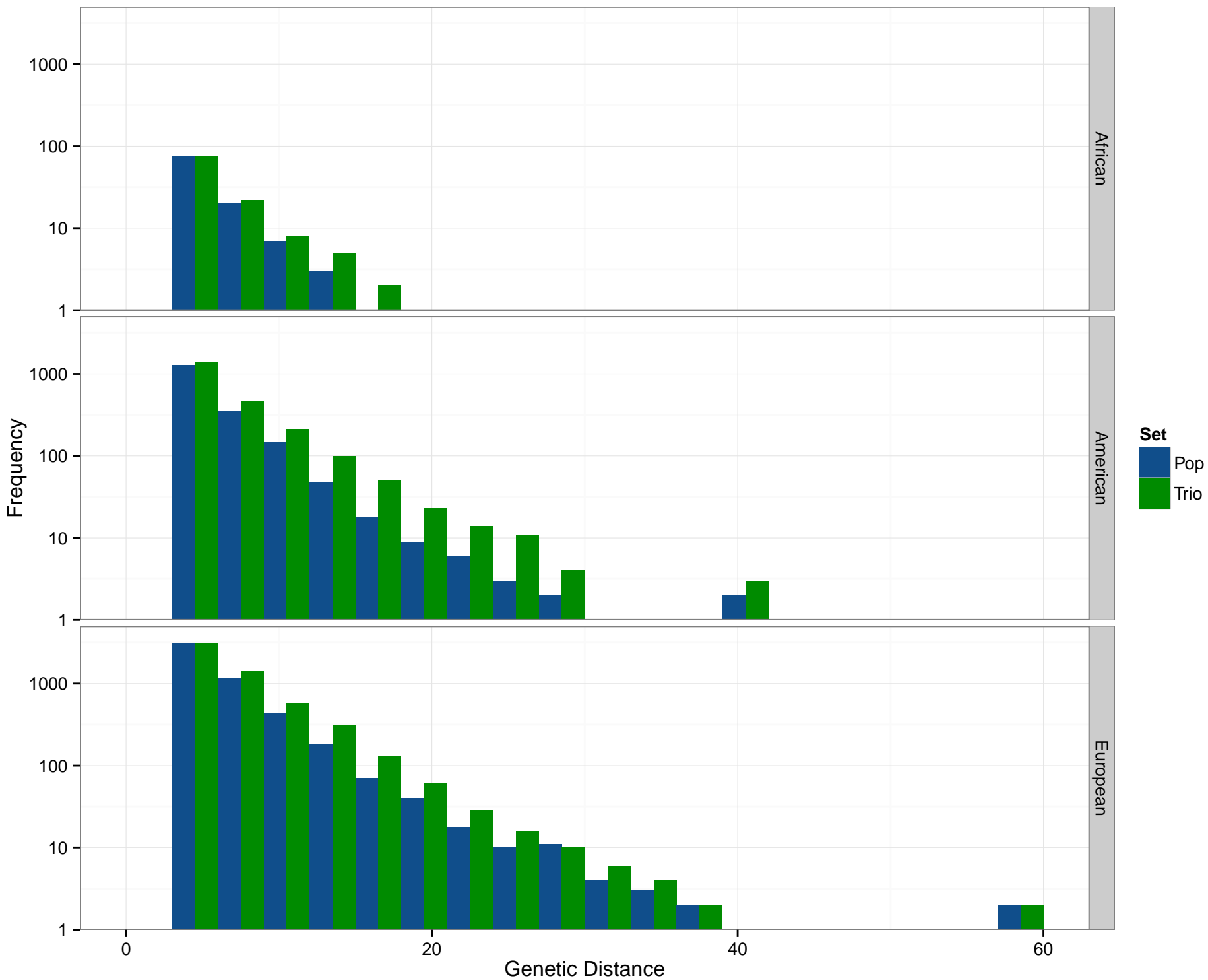

Supplement: S32 Fig — Data are shown on a log-scale to facilitate comparisons. (PDF) [file pgen.1005602.s032.pdf]
