# Supplementary material for: Genomic Insights into the Ancestry and Demographic History of South America
Source: PLoS Genet. 2015 Dec 4;11(12):e1005602. doi: 10.1371/journal.pgen.1005602 (PMC4670080; doi:10.1371/journal.pgen.1005602)
Supplement: S21 Fig — Comparison of the proportion of European ancestry in an IBD tract compared with IBD tract length for tracts shared within each individual population. (PDF) [file pgen.1005602.s021.pdf]

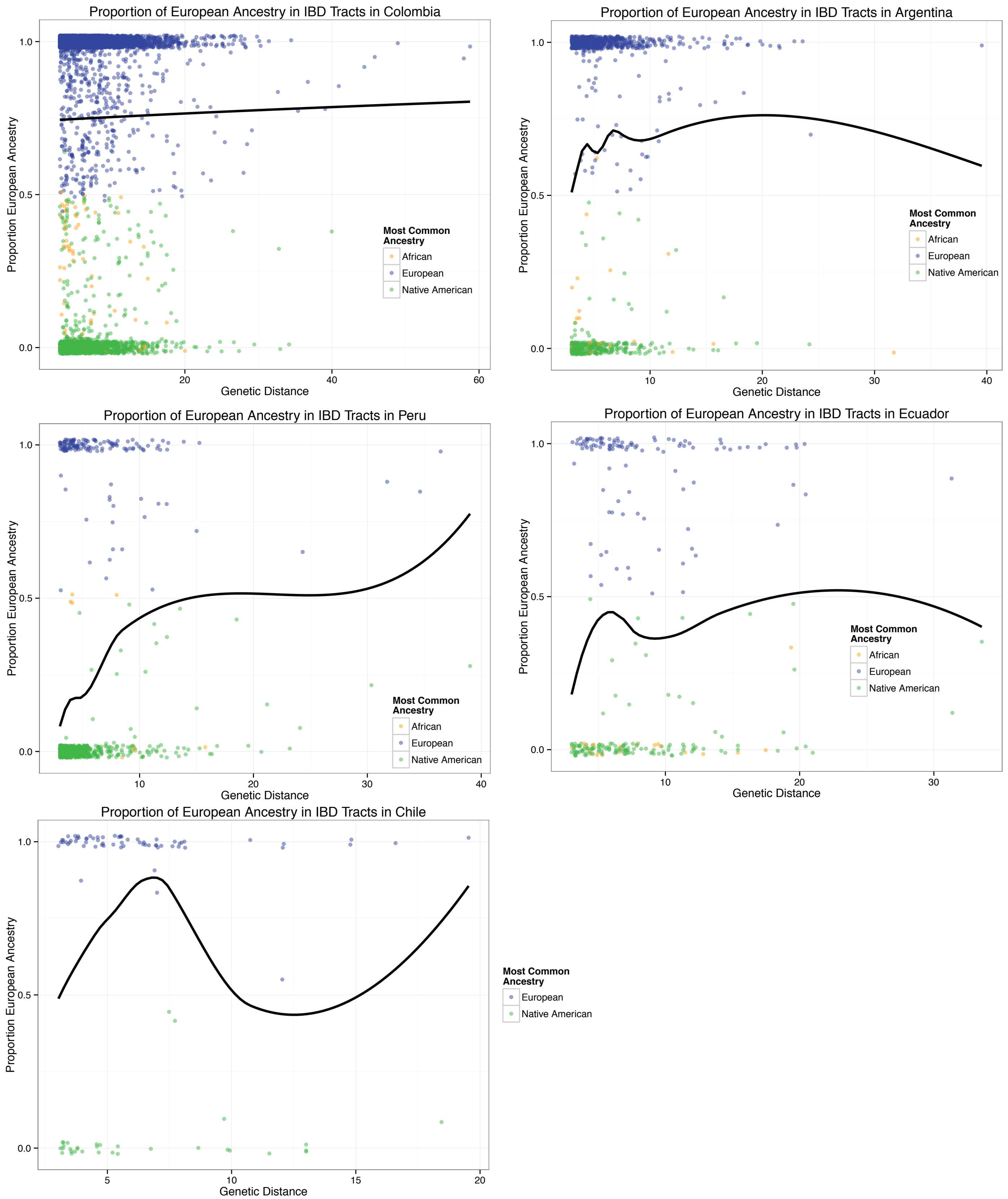
Proportion of European Ancestry in IBD Tracts in Colombia
Proportion European Ancestry
Genetic Distance
0.0
0.5
1.0
20
40
60
Most Common Ancestry
African
European
Native American
Proportion of European Ancestry in IBD Tracts in Argentina
Proportion European Ancestry
Genetic Distance
0.0
0.5
1.0
10
20
30
40
Most Common Ancestry
African
European
Native American
Proportion of European Ancestry in IBD Tracts in Peru
Proportion European Ancestry
Genetic Distance
0.0
0.5
1.0
10
20
30
40
Most Common Ancestry
African
European
Native American
Proportion of European Ancestry in IBD Tracts in Ecuador
Proportion European Ancestry
Genetic Distance
0.0
0.5
1.0
10
20
30
Most Common Ancestry
African
European
Native American
Proportion of European Ancestry in IBD Tracts in Chile
Proportion European Ancestry
Genetic Distance
0.0
0.5
1.0
5
10
15
20
Most Common Ancestry
European
Native American
