# Supplementary material for: Genomic Insights into the Ancestry and Demographic History of South America
Source: PLoS Genet. 2015 Dec 4;11(12):e1005602. doi: 10.1371/journal.pgen.1005602 (PMC4670080; doi:10.1371/journal.pgen.1005602)

Base Model

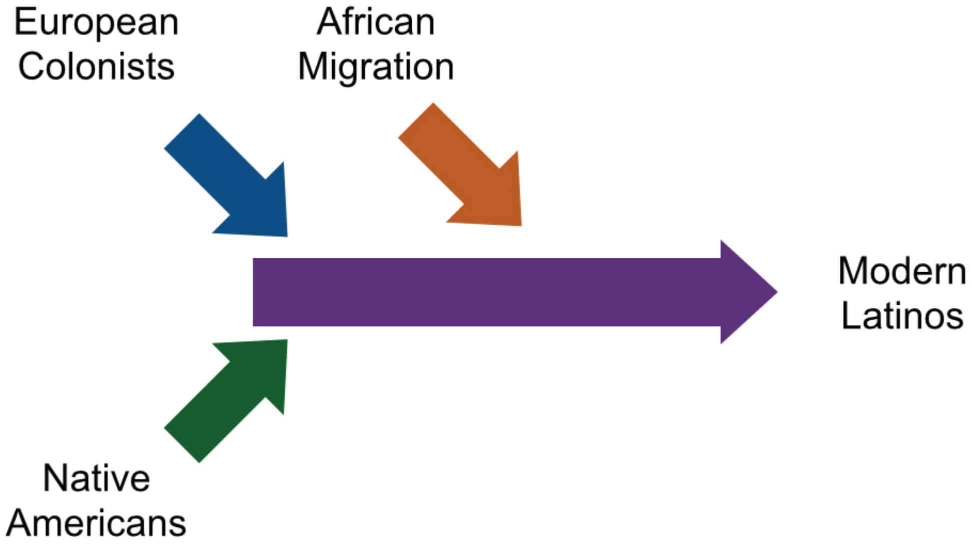

Model with Additional European Migration

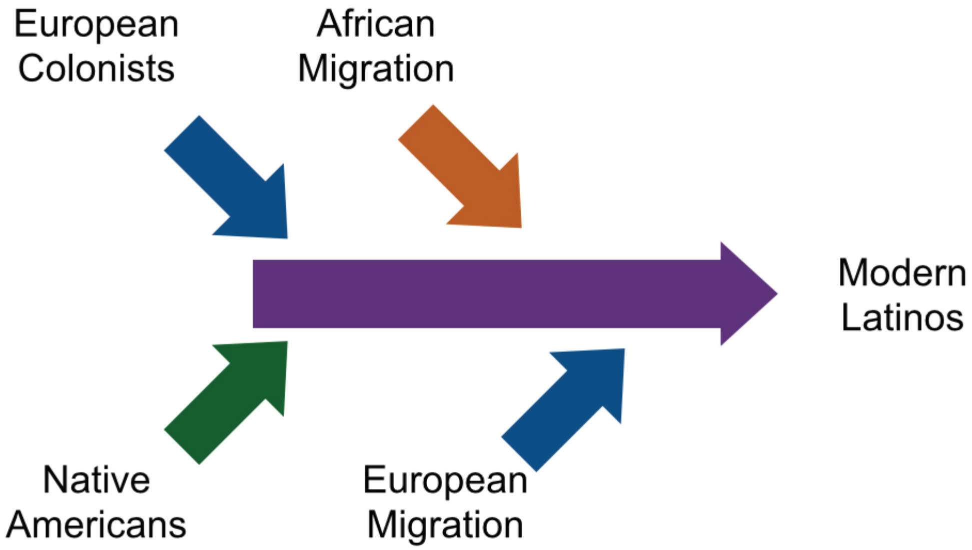

Model with Additional African Migration

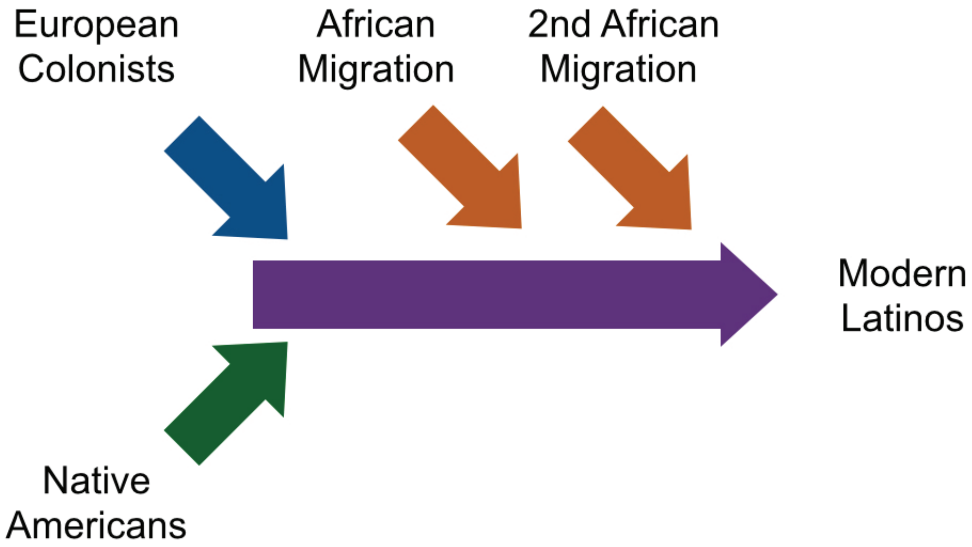

Supplement: S24 Fig — Schematic of the three models tested in Tracts. The top represents the base model, the second panel represents the models with an additional pulse of European migration, and the third panel represents the model with an additional pulse of African migration. (PDF) [file pgen.1005602.s024.pdf]
